# Undiagnosed RASopathies in infertile men

**DOI:** 10.3389/fendo.2024.1312357

**Published:** 2024-04-09

**Authors:** Anna-Grete Juchnewitsch, Kristjan Pomm, Avirup Dutta, Erik Tamp, Anu Valkna, Kristiina Lillepea, Eisa Mahyari, Stanislav Tjagur, Galina Belova, Viljo Kübarsepp, Helen Castillo-Madeen, Antoni Riera-Escamilla, Lisanna Põlluaas, Liina Nagirnaja, Olev Poolamets, Vladimir Vihljajev, Mailis Sütt, Nassim Versbraegen, Sofia Papadimitriou, Robert I. McLachlan, Keith A. Jarvi, Peter N. Schlegel, Sven Tennisberg, Paul Korrovits, Katinka Vigh-Conrad, Moira K. O’Bryan, Kenneth I. Aston, Tom Lenaerts, Donald F. Conrad, Laura Kasak, Margus Punab, Maris Laan

**Affiliations:** ^1^Chair of Human Genetics, Institute of Biomedicine and Translational Medicine, University of Tartu, Tartu, Estonia; ^2^Andrology Clinic, Tartu University Hospital, Tartu, Estonia; ^3^Centre of Pathology, East Tallinn Central Hospital, Tallinn, Estonia; ^4^Division of Genetics, Oregon National Primate Research Center, Oregon Health and Science University, Beaverton, OR, United States; ^5^Department of Surgery, Institute of Clinical Medicine, University of Tartu, Tartu, Estonia; ^6^Department of Pediatric Surgery, Clinic of Surgery, Tartu University Hospital, Tartu, Estonia; ^7^Interuniversity Institute of Bioinformatics in Brussels, Université Libre de Bruxelles-Vrije Universiteit Brussel, Brussels, Belgium; ^8^Machine Learning Group, Université Libre de Bruxelles, Brussels, Belgium; ^9^Department of Biomolecular Medicine, Faculty of Medicine and Health Science, Ghent University, Ghent, Belgium; ^10^Hudson Institute of Medical Research and the Department of Obstetrics and Gynecology, Monash University, Clayton, VIC, Australia; ^11^Division of Urology, Department of Surgery, Mount Sinai Hospital, University of Toronto, Toronto, ON, Canada; ^12^Department of Urology, Weill Cornell Medical College, New York, NY, United States; ^13^School of BioSciences, Faculty of Science, The University of Melbourne, Parkville, VIC, Australia; ^14^Andrology and IVF Laboratory, Department of Surgery (Urology), University of Utah School of Medicine, Salt Lake City, UT, United States; ^15^Artificial Intelligence Laboratory, Vrije Universiteit Brussel, Brussels, Belgium; ^16^Center for Embryonic Cell and Gene Therapy, Oregon Health and Science University, Beaverton, OR, United States

**Keywords:** cryptorchidism, syndromic male infertility, exome sequencing, RAS/MAPK pathway, molecular diagnosis, cancer, congenital testicular dysgenesis, multidisciplinary management

## Abstract

RASopathies are syndromes caused by congenital defects in the Ras/mitogen-activated protein kinase (MAPK) pathway genes, with a population prevalence of 1 in 1,000. Patients are typically identified in childhood based on diverse characteristic features, including cryptorchidism (CR) in >50% of affected men. As CR predisposes to spermatogenic failure (SPGF; total sperm count per ejaculate 0–39 million), we hypothesized that men seeking infertility management include cases with undiagnosed RASopathies. Likely pathogenic or pathogenic (LP/P) variants in 22 RASopathy-linked genes were screened in 521 idiopathic SPGF patients (including 155 CR cases) and 323 normozoospermic controls using exome sequencing. All 844 men were recruited to the ESTonian ANDrology (ESTAND) cohort and underwent identical andrological phenotyping. RASopathy-specific variant interpretation guidelines were used for pathogenicity assessment. LP/P variants were identified in *PTPN11* (two), *SOS1* (three), *SOS2* (one), *LZTR1* (one), *SPRED1* (one), *NF1* (one), and *MAP2K1* (one). The findings affected six of 155 cases with CR and SPGF, three of 366 men with SPGF only, and one (of 323) normozoospermic subfertile man. The subgroup “CR and SPGF” had over 13-fold enrichment of findings compared to controls (3.9% *vs.* 0.3%; Fisher’s exact test, *p* = 5.5 × 10^−3^). All ESTAND subjects with LP/P variants in the Ras/MAPK pathway genes presented congenital genitourinary anomalies, skeletal and joint conditions, and other RASopathy-linked health concerns. Rare forms of malignancies (schwannomatosis and pancreatic and testicular cancer) were reported on four occasions. The Genetics of Male Infertility Initiative (GEMINI) cohort (1,416 SPGF cases and 317 fertile men) was used to validate the outcome. LP/P variants in *PTPN11* (three), *LZTR1* (three), and *MRAS* (one) were identified in six SPGF cases (including 4/31 GEMINI cases with CR) and one normozoospermic man. Undiagnosed RASopathies were detected in total for 17 ESTAND and GEMINI subjects, 15 SPGF patients (10 with CR), and two fertile men. Affected RASopathy genes showed high expression in spermatogenic and testicular somatic cells. In conclusion, congenital defects in the Ras/MAPK pathway genes represent a new congenital etiology of syndromic male infertility. Undiagnosed RASopathies were especially enriched among patients with a history of cryptorchidism. Given the relationship between RASopathies and other conditions, infertile men found to have this molecular diagnosis should be evaluated for known RASopathy-linked health concerns, including specific rare malignancies.

## Introduction

RASopathies or RAS/mitogen-activated protein kinase (MAPK) syndromes are a group of phenotypically overlapping congenital syndromes caused by germline disease-causing variants in 22 genes encoding components of the Ras/MAPK pathway (1, 2). Dysfunction of this cellular signaling pathway during fetal development causes a spectrum of syndromes characterized by skeletal, cardiac, gastrointestinal, neurologic, and dermatological conditions and, on some occasions, distinct facial features. Cryptorchidism (CR), testicular maldescent, has been reported in ~60%–80% of male patients with Noonan syndrome (NS) (3, 4), the most prevalent RASopathy. CR due to primary testicular dysgenesis represents an established risk factor for spermatogenic failure (SPGF) in adulthood (5, 6).

There are limited data on reproductive and general health comorbidities in adults carrying germline variants in RASopathy-linked genes, as most studies have been conducted in pediatric cohorts. We recently reported an infertile man with bilateral CR and cryptozoospermia carrying a pathogenic variant in *SOS1* and proposed genetic alterations in the Ras/MAPK pathway as a novel etiology implicated in congenital CR and SPGF (7). We hypothesized that SPGF patients seeking infertility management include cases with undiagnosed RASopathies. This study aimed to screen a large cohort of unexplained SPGF cases compared to normozoospermic men for disease-causing variants in the Ras/MAPK pathway genes and to characterize the respective genotype–phenotype links in detail.

## Patients and methods

### Ethics statement

Research of the ESTonian ANDrology (ESTAND) cohort has been approved by the Ethics Review Committee of Human Research of the University of Tartu, Estonia (permissions no. 74/54 and 118/69 with last amendment 288/M-13; 221/T-6, 286/M-18). All ESTAND study subjects have been recruited at the Andrology Clinic of Tartu University Hospital (AC-TUH), managing >90% of all severe male infertility cases in Estonia (5). Research in the involved Genetics of Male Infertility Initiative (GEMINI) study centers has been approved by IRB_00063950 (IRB of the University of Utah, USA); 16030459 and 0102004794 (IRB of Weill Cornell Medical College, New York, USA); 15-0147-E (IRB of Mount Sinai Hospital, University of Toronto, Toronto, Canada); and human ethics committees of Monash Surgical Day Hospital, Monash Medical Centre and Monash University, Australia.

Written informed consent was obtained from each patient before recruitment. The study was carried out in compliance with the Declaration of Helsinki.

### ESTAND cohort study group formation

The study group for whole-exome sequencing (WES) consisted of 844 participants of the ESTAND cohort (PI: M. Punab and M. Laan): 521 patients with idiopathic SPGF and 323 normozoospermic partners of pregnant women as controls (CTRL; **Table 1**) (5, 9). All men had undergone identical routine andrological workup using the established clinical pipeline and standard protocols at the AC-TUH (**Supplementary Methods**). SPGF was defined as a total sperm count of 0–39 million per ejaculate (8). Patients were prioritized to WES based on severe unexplained SPGF suggesting a possible genetic cause (e.g., no sperms or extremely low total sperm count per ejaculate, abnormal hormonal parameters, and genitourinary conditions). Men with at least one testicle missing in the scrotum at the recruitment or medical history of CR formed the “SPGF with CR” subgroup (n = 155), whereas the rest were defined as the “SPGF only” subgroup (n = 366). The two patient subgroups did not differ in andrological parameters, except for lower total testis volume (TTV) in CR cases (24.0 *vs.* 27.5 mL, *p* = 5.71 × 10^−5^; **Table 1**). “SPGF only” patients (median age 35.4 y) were slightly older than “SPGF with CR” (32.4 y) and CTRL men (31.0 y, *p* < 0.001), and normozoospermic men had lower body mass index (BMI) than patients (25.0 *vs.* 26.2–26.6, *p* < 0.05).

**Table 1:** Clinical parameters of the ESTAND study group.

Parameters	SPGF with CR^a^	SPGF only	CTRL^b^
Subjects (n)	155	366	323
Age (y)	32.4 (21.8–52.7)	35.4 (23.6–52.6)*	31.0 (23.0–45.0)
Height (cm)	181 (164–192)	180 (169–193)	180 (171–191)
Weight (kg)	85.3 (62.6–137.6)	87.1 (65.0–116.8)	82.3 (64.0–107.9)
BMI (kg/m^2^)	26.2 (20.5–39.1)	26.6 (20.3–35.7)	25.0 (19.9–32.3)*
Sperm count (×10^6^/ejaculate)	0.0 (0.0–7.6)	0.0 (0.0–9.9)	303.1 (70.3–978.2)
Sperm conc. (×10^6^/mL)	0.0 (0.0–2.5)	0.0 (0.0–3.5)	80.0 (20.0–244.6)
Sperm motility (A + B, %)	11.5 (0.0–59.4)	18.5 (0.0–63.0)	50.0 (28.0–70.0)
Sperm morphology (%)	0.0 (0.0–8.3)	0.0 (0.0–5.0)	10.0 (2.0–20.0)
Semen volume (mL)	3.1 (0.0–8.0)	3.3 (0.0–6.5)	3.7 (1.8–8.0)
FSH (IU/L)	21.6 (4.2–60.2)	18.0 (6.3–47.2)	3.5 (1.4–8.2)
LH (IU/L)	9.3 (3.1–24.7)	8.5 (3.3–19.2)	3.6 (1.5–6.7)
Testosterone (nmol/L)	14.2 (3.9–27.8)	14.6 (6.1–30.0)	16.1 (7.9–26.9)
PSA (ng/mL)	0.6 (0.0–1.5)	0.6 (0.2–1.8)	0.7 (0.3–1.4)
TV, left (mL)	12.0 (0.0–24.3)	13.0 (7.0–23.0)	23.0 (16.9–30.0)
TV, right (mL)	13.0 (0.0–24.0)	15.0 (6.0–24.0)	24.0 (18.0–35.0)
TV, total (mL)	24.0 (3.0–42.0)#	27.5 (13.3–47.0)#	46.0 (34.0–63.0)
Total TV < 30 mL (n, %)	106/155 (68.4%)	212/366 (57.9%)	8/323 (2.5%)
Cryptorchidism (n, %)	155 (100%)	0 (0.0%)	8 (2.5%)^c^
Hypospadias (n, %)	8 (5.2%)	7 (1.9%)	2 (0.6%)

Data are shown as median (5–95%), unless indicated otherwise. Andrological parameters between the three subgroups differed according to their formation criteria.

BMI, body mass index; conc., concentration; ESTAND, ESTonian ANDrology; FSH, follicle-stimulating hormone; LH, luteinizing hormone; TV, testis volume; PSA, prostate-specific antigen; y, years.

Reference values: total sperm count >39 × 10^6^/ejaculate; sperm concentration >16 × 10^6^/mL; sperm motility >32%; sperm morphology >4%; semen volume >1.5 mL; FSH 1.5–12.4 IU/L; LH 1.7–8.6 IU/L; testosterone 8.64–29.0 nmol/L (19–49 y) and 6.68–25.7 nmol/L (≥50 y); PSA <1.4 ng/mL (<40 y), <2.0 ng/mL (40–49 y), <3.1 ng/mL (50–59 y), <4.1 ng/mL (60–69 y), and <4.4 ng/mL (≥70 y).

a Spermatogenic failure (SPGF) was defined as total sperm count of 0–39 million per ejaculate (8); cryptorchidism (CR) refers to at least one testicle missing in the scrotum at the recruitment or medical history of CR (orchidopexy or spontaneous descent).

b Control (CTRL) group was formed from normozoospermic (total sperm count >39 million/ejaculate) partners of pregnant women (5, 9).

c Normozoospermic men with proven fatherhood, but one undescended testicle at the time of recruitment (three subjects) or medical history of CR resolved by spontaneous descent (two) or orchidopexy (three) during childhood.

* Significant difference compared to other clinical groups (Mann–Whitney *U* test, p < 0.05).

# The two patient subgroups differed in total TV (p = 5.71 × 10^−5^).

Eight of 10 subjects with disease-causing findings in the Ras/MAPK pathway genes consented to a follow-up assessment based on the established clinical recommendations for the phenotyping of RASopathy patients (10, 11) (**Supplementary Methods**).

### Disease-causing variant detection in the WES dataset

WES data were generated in three next-generation sequencing service laboratories: the Institute for Molecular Medicine Finland (FIMM), Helsinki, Finland (n = 447) (7, 12), the McDonnell Genome Institute of Washington University in St. Louis, MO, USA (n = 82) as previously described (13, 14), and the Huntsman Cancer Institute High-Throughput Genomics Core Facility at the University of Utah, Salt Lake City, UT, USA (n = 315) (**Supplementary Methods**).

VCF files generated by all laboratories were filtered for variant quality using identical parameters (exclusion DP < 10 and GQ < 20) and merged into a single VCF file to be annotated using the Ensembl platform tool VEP v105 (15) (**Supplementary Table 1**). Likely pathogenic (LP) or pathogenic (P) variants were screened in 22 Ras/MAPK pathway genes, including 20 autosomal dominant (AD) genes, *SPRED2* linked to autosomal recessive, and *LZTR1* linked to both modes of inheritance (**Supplementary Table 2**). VEP output files were subjected to three stages of filtering: custom-designed pipeline for automatic exclusion of variants with an unlikely monogenic disease-causing effect (**Supplementary Table 3**), variant pathogenicity interpretation with the AI-based tool (https://franklin.genoox.com—Franklin by Genoox) to exclude likely benign and benign predictions, and final manual classification of the retained variants using the American College of Medical Genetics and Genomics (ACMG) guidelines for the Ras/MAPK pathway genes (16) (**Supplementary Table 5**).

All 10 subjects with findings in the Ras/MAPK gene panel underwent extended WES data analysis to assess whether the identified LP/P variant was a monogenic cause explaining the patient’s phenotype. The ORVAL (Oligogenic Resource for Variant AnaLysis) platform (17) and VarCoPP2.0 tool (18) were implemented for statistical modeling and estimating the probability of digenic pathogenicity. Further details of WES data processing and analysis are provided in **Supplementary Methods**.

### Testicular histopathology and immunohistochemical staining

Testis biopsies were collected, and histopathological examinations were carried out for 110 of 521 (21.0%) SPGF cases at the Pathology Centre, Diagnostic Clinic of the East Tallinn Central Hospital, Estonia, or Pathology Department, Tartu University Hospital, Estonia, following the guidelines (19). In the clinical setting, this invasive procedure was undertaken only when justified for clinical decision making in infertility management and consented by the patient.

Immunohistochemistry was applied to analyze PTPN11 protein expression in paraffin-embedded testis biopsy samples from Case 1 with PTPN11 p.N308D (35 y) and a control subject (38 y, obstructive azoospermia, histologically normal active spermatogenesis). The monoclonal rabbit anti-SHP2 (Y478) antibody (Abcam, Cambridge, UK) was diluted 1:10 in Antibody Diluent (Dako, Agilent, Santa Clara, USA) and incubated for 60 minutes at 37°C. The antigen–antibody complex was visualized using the Ventana OptiView DAB IHC Detection and Amplification Kits and Reaction Buffer for washing steps (Roche Diagnostics GmbH, Mannheim, Germany). Details are reported in **Supplementary Methods.**

### Validation cohort for the assessment of RASopathy-linked variants in infertile men

Further assessment for RASopathy-linked variants in SPGF patients was performed in the WES dataset of the GEMINI cohort (PI: D.F. Conrad and K.I. Aston). This cohort has been mainly formed to discover novel monogenic causes of non-obstructive azoospermia (NOA) among idiopathic cases recruited in andrology clinics worldwide (13, 14) (**Supplementary Methods**). Assessment for LP/P variants in 22 RASopathy-linked genes in the GEMINI cohort was carried out as described above. Variants were screened in 1,416 SPGF patients (1,119 NOA and 297 oligozoospermia cases) and 317 fertile controls (**Supplementary Table 6**). For 31 SPGF cases, medical history of cryptorchidism had been documented. Variant filtering and interpretation followed an identical pipeline as utilized for the ESTAND data. The final manual classification of the retained variants was carried out according to the ACMG guidelines (16) (**Supplementary Table 7**).

### Experimental confirmation of the predicted LP/P variants

All variants predicted to be disease-causing based on the WES data analysis in the ESTAND and GEMINI cohorts were confirmed by Sanger sequencing (**Supplementary Figures 1–3**, **Supplementary Table 4**).

### Testicular gene expression dataset

Gene expression levels in distinct testicular cell types in six adult control patients were derived from the human testis single-cell RNA sequencing (scRNA-seq) dataset curated and available at the human infertility single-cell testis atlas (HISTA ver. 2.9.6) (20). As previously described, the gene expression used for this manuscript is the batch-corrected normalized log counts.

## Results

### Disease-causing variants in RASopathy-linked genes

**Figure 1:**
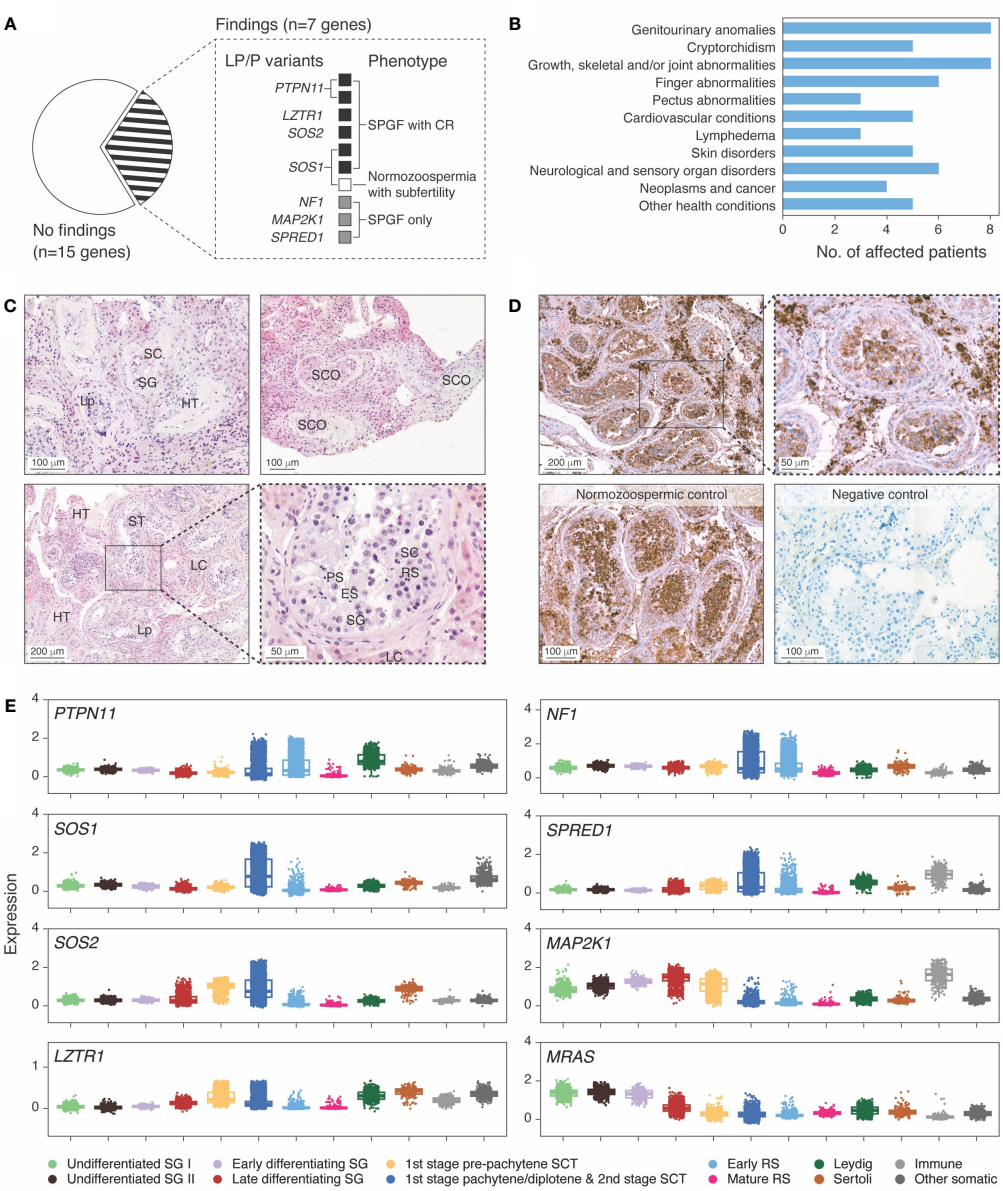
Ras/mitogen-activated protein kinase (MAPK) signaling pathway genes with disease-causing variants in idiopathic male infertility patients. **(A)** Distribution of identified likely pathogenic (LP) and pathogenic (P) variants across genes and clinical phenotypes. Spermatogenic failure (SPGF) was defined as total sperm count of 0–39 million per ejaculate (8). Cryptorchidism (CR) refers to at least one testicle missing in the scrotum at the recruitment or medical history of testicular maldescent. A subfertile normozoospermic man with SOS1 p.I437T presented low-normal sperm count (50 million per ejaculate) and needed infertility management to achieve pregnancy. **(B)** Prevalent health conditions in eight (of 10 total) idiopathic SPGF patients carrying LP/P variants in Ras/MAPK pathway genes available for the follow-up clinical assessment. **(C)** Testicular histology of Case 1 carrying *PTPN11* c.922A>G (p.N308D) variant showed mixed testicular atrophy and heterogeneous morphology. Most seminiferous tubules (ST) were narrowed due to thickened lamina propria (Lp), and ~30% were completely or partially hyalinized (HT). ST with Sertoli cells (SC) and spermatogonia (SG) represented <10%, and those with Sertoli cells only (SCO) represented 20%. Maturation arrest in the primary (PS) or secondary spermatocyte stage was observed in ~10%. In the rest of ST, all spermatogenic stages were detected, but the number of round spermatids (RS) and elongated spermatids (ES) was reduced. Severe Leydig cell (LC) hyperplasia was noticeable in the stroma surrounding the tubules. Details are provided in **Supplementary Table 8**. **(D)** Immunohistochemical staining of PTPN11 in testicular tissue sections in Case 1 with the PTPN11 p.N308D substitution and in a control case with histologically normal active spermatogenesis (38 y, obstructive azoospermia). PTPN11 protein localized in the cytoplasm of spermatogenic cells, LC and SC. Staining intensity in the control case was the strongest in primary spermatocytes, early round spermatids, and LC, whereas in spermatogonia and SC, only faint PTPN11 positivity was detected. In Case 1 tissue, the strongest staining was detected in LC, consistent with the testicular histology **(C)**. In the negative control performed without the PTPN11 antibody, no staining was detected. Brown color indicates chromogen-labeled antibody, and violet color indicates hematoxylin background staining. **(E)** Testicular mRNA expression of genes with identified LP/P variants in SPGF patients. Normalized log gene expression levels (Y-axis) in distinct testicular cell types were derived from the human infertility single-cell testis atlas (HISTA ver. 2.9.6) (20). The category “immune cells” includes lymphoid T cells and rare B cells as well as myeloid M1/M2 macrophages. The “other cells” category comprises endothelial and myoid cells. Each dot represents data from one cell. Boxes include 25%–75% of the data points, and the line shows median expression. SCT, spermatocytes; RS, round spermatids.

Nine SPGF patients and one CTRL subject in the ESTAND cohort carried heterozygous LP/P variants in seven of 22 RASopathy-linked genes (**Figure 1A**, **Table 2**). All but one variant (SPRED1 p.R325Ter) were missense substitutions. Five of 10 findings were reported in the ClinVar database as LP/P variants linked to NS or Legius syndrome, including two well-established substitutions, PTPN11 p.N308D and p.M504V [Food and Drug Administration (FDA) expert panel: P]. Three variants were novel and two were ultra-rare, not reported in the worldwide population-based Genome Aggregation Database (gnomAD). These were classified as LP according to the guidelines (16) and supported by extended patient phenotyping and family health history.

**Table 2:** Clinical characteristics of patients with RASopathy-linked variants in the ESTAND cohort.

Case (age)^a^	Variant data^b^	H W BMI	Andrological data^c^	Other health data	Family health history
1 (35/49 y)	*PTPN11* c.922A>G p.N308D (P) #* rs28933386 MAF 1.2 × 10^−5^	163 cm 59 kg 22.2	CR (bil), op. at 8 y; shrinking of the right testis after orchidopexy. TV 12 + 10 = 22 mL FSH 57.7 IU/L LH 20.6 IU/L T 22.3 nmol/L NOA, TESA negative. Testis histology: **Figure 1C**, **Supplementary Table 8**.	Postnatal growth restriction (120 cm at 14 y); reduced pubertal growth spurt, GH therapy to reach current height. Pectus carinatum, camptodactyly, heart arrhythmia, aortic valve defect, celiac disease, hepatomegaly; muscle hypotonia, hypoplasia of abdominal wall muscles; hyperthyroidism, keratosis pilaris, widely spaced nipples. Facial features typical of NS: triangular face, midface retrusion, prominent forehead, micrognathia; dental malocclusion; ocular hypertelorism and proptosis, severe ptosis since birth, epicanthus, strabismus, lacrimal gland anomalies; wide-based depressed nose with bulbous upturned tip, deep philtrum, prominent nasolabial folds; low-set backward-rotated ears with thick helix; short, broad, and webbed neck. Normal intelligence and cognition.	Most members tend to be tall, including father; two tall and fertile brothers. Given the family history, the patient was suspected to carry a *de novo* mutation.
2 (36/43 y)	*PTPN11* c.1510A>G p.M504V (P) #* rs397507547 MAF 4.0 × 10^−6^	158 cm 60 kg 24.0	CR (bil), op. at 4 y TV 12 + 14 = 26 mL FSH 48.0 IU/L LH 11.3 IU/L T 12.8 nmol/L NOA, TESA negative.	The patient is a single child. Postnatal growth restriction; delayed puberty and pubertal growth spurt. Pectus excavatum (left), short fingers. Sensitive digestive system; muscle hypotonia, hearing impairment, severe migraine, numbness of hands, keratosis pilaris, widely spaced nipples. Facial features typical of NS: large head, curly hair, ocular hypertelorism, wide-based depressed nose with bulbous upturned tip, low-set backward-rotated ears, short neck. Learning difficulties, dyslexia, dysgraphia. Normal cognition.	Father: height <160 cm, spine surgery, physical disability since 50 y. Uncle (pat): childless, mental illness, and lifelong hospitalization.
3 (31/35 y)	*SOS1* c.642A>C p.Q214H (LP) Novel variant	175 cm 81 kg 26.4	CR (uni), op. at 7 y TV 9 + 15 = 24 mL FSH 40.5 IU/L LH 12.6 IU/L T 15.6 nmol/L Total sperm count per ejaculate 0.6 × 10^6^	The patient is a single child. Seminoma and germ cell neoplasia *in situ* (diagnosis at 33 y). Short fingers, joint stiffness in knees; severe dental malocclusion, gingival bleeding, complications (op. at 17 y), myopia. No typical facial features of NS except for a large head and prominent forehead. Normal intelligence and cognition.	Family (mat): several childless men with early mortality. Mother and grandfather (mat): joint stiffness.
4 (29 y/n.a.)	*SOS1* c.406T>C p.Y136H (LP) Novel variant	194 cm 99 kg 26.3	CR (bil) TV 20 + 7 = 27 mL FSH 16.6 IU/L LH 7.8 IU/L T 10.9 nmol/L Total sperm count per ejaculate 0.9 × 10^6^	Erythema, multiple nevi. A previously reported case (7) recruited to the ESTAND cohort and published in a study of a single family with a spectrum of reproductive system phenotypes. Not available for follow-up assessment.	Father and uncle (pat): prostate cancer.
5 (30 y/n.a.)	*SOS1* c.1310T>C p.I437T (LP) # rs397517150 MAF n.a.	173 cm 75 kg 25.1	TV 12 + 15 = 27 mL FSH 12.4 IU/L LH 4.9 IU/L T 25.6 nmol/L Total sperm count per ejaculate 50.0 × 10^6^	Subfertility, time to pregnancy >2 y, one child with supportive infertility management. Varicocele sin grade 3. Not available for follow-up assessment.	Not available.
6 (27/33 y)	*SOS2* c.26A>G, p.E9G (LP) Novel variant/*KIF7* c.434A>C, p.Y145S (LP) rs758361736 MAF 1.3 × 10^−5^/*CHEK1* c.1036C>T, p.Q346Ter (LP) rs199535573 MAF 3.9 × 10^−5^	178 cm 85 kg 26.8	CR (uni), op. at 1 y TV 24 + 0 = 24 mL FSH 11.9 IU/L LH 6.1 IU/L T 6.2/4.3 nmol/L Progressing hypogonadism. Anejaculation	Premature birth, developmental delay. Scoliosis, camptodactyly, cubitus valgus; spastic diplegic cerebral palsy and spastic paraplegia, epilepsy since childhood; severe lymphedema of both lower legs. Facial features typical of NS: large head, high and wide forehead, sparse fine hair and alopecia, ptosis, wide-based depressed nose with bulbous upturned tip, deep philtrum, micrognathia, low-set backward-rotated ears with thick helix, short and broad neck, ocular hypertelorism, strabismus, acne. Impaired cognition and intellectual performance, reduced eye contact, severe articulation difficulties, psychiatric conditions, and special educational needs. Died at 33 y (pancreatic cancer).	See **Figure 2A**.
7 (38/55 y)	*LZTR1* c.848G>A p.R283Q (P) # rs1223430276 MAF n.a.	182 cm 73 kg 22.0	CR (uni), op. at 8 y TV 3 + 4 = 7 mL FSH 62.0 IU/L LH 10.2 IU/L T 17.8 nmol/L NOA Histology: SCOS	Delayed growth spurt (at 16 y), problems with digestion in childhood; mild pectus excavatum, heart arrhythmia, congenital one-sided impaired vision and hearing, migraine; schwannomatosis; paralysis of vocal cords and larynx. No typical facial features of NS. Childhood dysarthria. Normal intelligence and cognition.	Father: heart problems, sudden death at 42 y. Aunt (pat): childless.
8 (48/54 y)	*NF1* c.4348G>T p.A1450S (LP) MAF n.a.	177 cm 90 kg 28.8	TV 8 + 12 = 20 mL FSH 36.3 IU/L LH 9.8 IU/L T 12.2 nmol/L Total sperm count per ejaculate 8.4 × 10^6^ Benign prostate hyperplasia	Scoliosis, several tibial bone fractures, gonarthrosis, prone to traumas, fifth finger clinodactyly, contracture of joints; neuralgia, lower limb lymphedema (from knees to feet), periodontitis; astigmatism, myopia; hypertension. Coarse facial features, wide-based depressed nose with bulbous upturned tip; ptosis and xanthelasma near both lower eyelids; alopecia (curly hair in youthhood). Normal intelligence and cognition. Natural conception at 27 y: 1 child.	Brother: pulmonary thrombosis, sudden death at 46 y. Niece: breast cancer, d. 37 y. Mother: dementia, fifth finger clinodactyly.
9 (26/35 y)	*SPRED1* c.973C>T p.R325Ter (P) # rs1057518683 MAF n.a./*TP63* c.1283C>T p.P428L (LP) Novel variant^d^	193 cm 101 kg 27.1	TV 25 + 25 = 50 mL FSH 15.2 IU/L LH 12.9 IU/L T 23.3 nmol/L Total sperm count per ejaculate 6.3 × 10^6^ Varicocelectomy	*Café-au-lait* macules, including large spots on the back and head. Vertebral defects, Schmorl’s nodes, congenital urethral stenosis (op. at 30 y), hydronephrosis, hypertension. No typical facial features of NS. Normal intelligence and cognition. Infertility treatment resulting in three natural conceptions: one deceased newborn (6 days after birth) with esophageal atresia, heart, and digit malformations; two children with no apparent developmental problems.	See **Figure 2B**.
10 (42/57 y)	*MAP2K1* c.635G>C p.S212T (LP) rs1019098903 MAF n.a.	180 cm 89 kg 27.5	TV 10 + 12 = 22 mL FSH 36.7 IU/L LH 10.0 IU/L T 15.7 nmol/L Total sperm count per ejaculate 0.03 × 10^6^ Histology: tubular hyalinization	Schwannomatosis (knees and arms), lymphedema (armpits, neck), multiple nevi, sparse fine hair; dental malocclusion. Short fingers, numbness of hands, hearing impairment (high-pitched sounds), migraine. No typical facial features of NS. Normal intelligence and cognition. Natural conception at 26 y: 1 child; ART management in his 40s: 1 singleton and twins.	Mother: schwannomatosis, frequent neoplasms, and uterine cysts (op.). Uncle (mat): cancer (d. in his 60s).

Details of the variant pathogenicity assessment are provided in **Supplementary Table 5**. Confirmation of variants by Sanger sequencing is shown in **Supplementary Figures 1, 2**.

ART, assisted reproductive technology; bil, bilateral; BMI, body mass index; BW, birth weight; CR, cryptorchidism; d., died; DD, developmental delay; ESTAND, ESTonian ANDrology; FSH, follicle-stimulating hormone; GH, growth hormone; H, height; het, heterozygous; LH, luteinizing hormone; LP, likely pathogenic; mat, maternal; n.a., not available; NOA, non-obstructive azoospermia; NS, Noonan syndrome; op., operated; P, pathogenic; pat, paternal; SCOS, Sertoli cell-only syndrome; T, testosterone; TESA, testicular sperm aspiration; TV, testis volume (left + right = total); uni, unilateral; W, weight; y, years.

a Age at the recruitment/at the time of the interview.

b Gene, cDNA and protein position, rs number, minor allele frequency (MAF; gnomAD v.2.1.1); LP/P variants reported in ClinVar (#) and reviewed by an expert panel (*); all reported RASopathy-linked variants were heterozygous, and pathogenicity was evaluated based on the ClinGen RASopathy Expert Panel guidelines (16); the pathogenicity of CHEK1, KIF7, and TP63 variants was evaluated using general ACMG guidelines (23).

c Hormonal profile at the recruitment. Reference values: FSH 1.5–12.4 IU/L (>18 y); LH 1.7–8.6 IU/L (>18 y); T 8.64–29.0 nmol/L.

d Incidental finding.

Differently from other affected RASopathy genes with the established dominant effect of LP/P variants, *LZTR1* has been linked to both dominant and recessive forms of NS (24). A previously reported dominant missense substitution LZTR1 p.R283Q (25) was found in an ESTAND patient presenting CR and NOA [Sertoli cell-only syndrome (SCOS)], as well as several characteristics of NS (pectus abnormalities, heart condition, schwannomatosis, and impaired vision and hearing).

### RASopathy-linked variants are overrepresented in the “SPGF with CR” subgroup

All 10 ESTAND subjects carrying LP/P variants in RASopathy-linked genes presented congenital genitourinary anomalies (**Figure 1B**, **Table 2**). Reduced TTV (<30 mL) was detected in all men except for Case 9 with a *SPRED1* truncating variant. The only RASopathy-linked variant identified among the CTRL subjects was SOS1 p.I437T (ClinVar: LP) detected in a man with low-normal total sperm count (50 million per ejaculate), reduced TTV (27 mL), and subfertility requiring supportive management for several years to conceive a child. He had no documented history of cryptorchidism and was unavailable for further clinical assessment. The SOS1 p.I437T variant has been previously reported in familial and sporadic NS cases (26).

The subgroup of “SPGF with CR” showed significant, 13-fold and fivefold enrichment of undiagnosed RASopathy cases (six of 155, 3.9%) compared to men with normozoospermia (one of 323, 0.3%; Fisher’s exact test, *p* = 5.5 × 10^−3^) and “SPGF only” cases (three of 366, 0.8%; *p* = 0.02, **Table 3**). Cryptorchid SPGF patients carried LP/P variants in classical NS genes *PTPN11*, *SOS1*, *SOS2*, or *LZTR1*, and all presented extremely low total sperm count (0.0–0.9 × 10^6^ per ejaculate). Testicular histology data were available for Case 1 with bilateral CR and NOA carrying PTPN11 p.N308D. Mixed testicular atrophy, thickened and hyalinized lamina propria of seminiferous tubules, reduced number of mature spermatids, and severe Leydig cell hyperplasia were observed (**Figure 1C**, **Supplementary Table 8**). Disease-causing variants in other Ras/MAPK pathway genes (*NF1*, *SPRED1*, and *MAP2K1*) were detected in patients with severe oligozoospermia without CR. These men had achieved fatherhood using their own gametes either at a young age and/or through assisted reproduction.

**Table 3:** Distribution of LP/P variants in 22 RASopathy-linked genes in the ESTAND subgroups.

Compared subjects	LP/P variant carriers (n, %)	OR [95%CI]	*p*-value ^a^
ESTAND study subgroups^b^			
SPGF with CR *vs.* CTRL	6/155 (3.9%) *vs.* 1/323 (0.3%)	12.9 [1.5–596.4]	5.5 × 10^−3^
SPGF with CR *vs.* SPGF only	6/155 (3.9%) *vs.* 3/366 (0.8%)	4.9 [1.0–30.4]	2.3 × 10^−2^
SPGF only *vs.* CTRL	3/366 (0.8%) *vs.* 1/323 (0.3%)	2.7 [0.2–140.0]	>0.05
All SPGF *vs.* CTRL	9/521 (1.7%) *vs.* 1/323 (0.3%)	5.7 [0.8–248.6]	>0.05

ESTAND, ESTonian ANDrology cohort; LP, likely pathogenic (LP); P, pathogenic.

a Fisher’s exact test; CI, confidence interval; OR, odds ratio.

b All ESTAND cohort patients with spermatogenic failure (SPGF), n = 521; SPGF with cryptorchidism (CR), n = 155; SPGF only, n = 366; control group of normozoospermic men (CTRL), n = 323.

### The spectrum of clinical manifestations in men with RASopathy-linked variants

Eight out of 10 ESTAND carriers of LP/P variants in the Ras/MAPK pathway genes were available for a follow-up assessment (**Figure 1B**, **Table 2**). Classical NS-related facial features and the highest number of congenital comorbidities were observed in patients with *PTPN11* and *SOS2* variants. All eight patients presented genitourinary anomalies and congenital skeletal and joint conditions. Other observed disorders affected skin, cardiac and circulatory, neurological, and sensory organ systems. Importantly, four of eight patients with follow-up data were diagnosed in early adulthood with rare forms of malignancies—schwannomatosis (*LZTR1* and *MAP2K1*), pancreatic cancer (*SOS2*), and seminoma and germ cell neoplasia *in situ* (*SOS1*). Only PTPN11 p.N308D and p.M504V variant carriers presented severe postnatal growth restriction and short stature (<3rd percentile), whereas the rest of the men had reached normal height. The onset of puberty had been normal in all but the patient with PTPN11 p.M504V.

The patient carrying the SOS2 p.E9G variant presented severe developmental delay (DD) with impaired cognitive and intellectual performance (*details below*). The rest of the men with RASopathy-linked variants had normal intelligence and comprehension, and half of them had received higher education.

### Two ESTAND patients with more than one disease-causing variant

**Figure 2:**
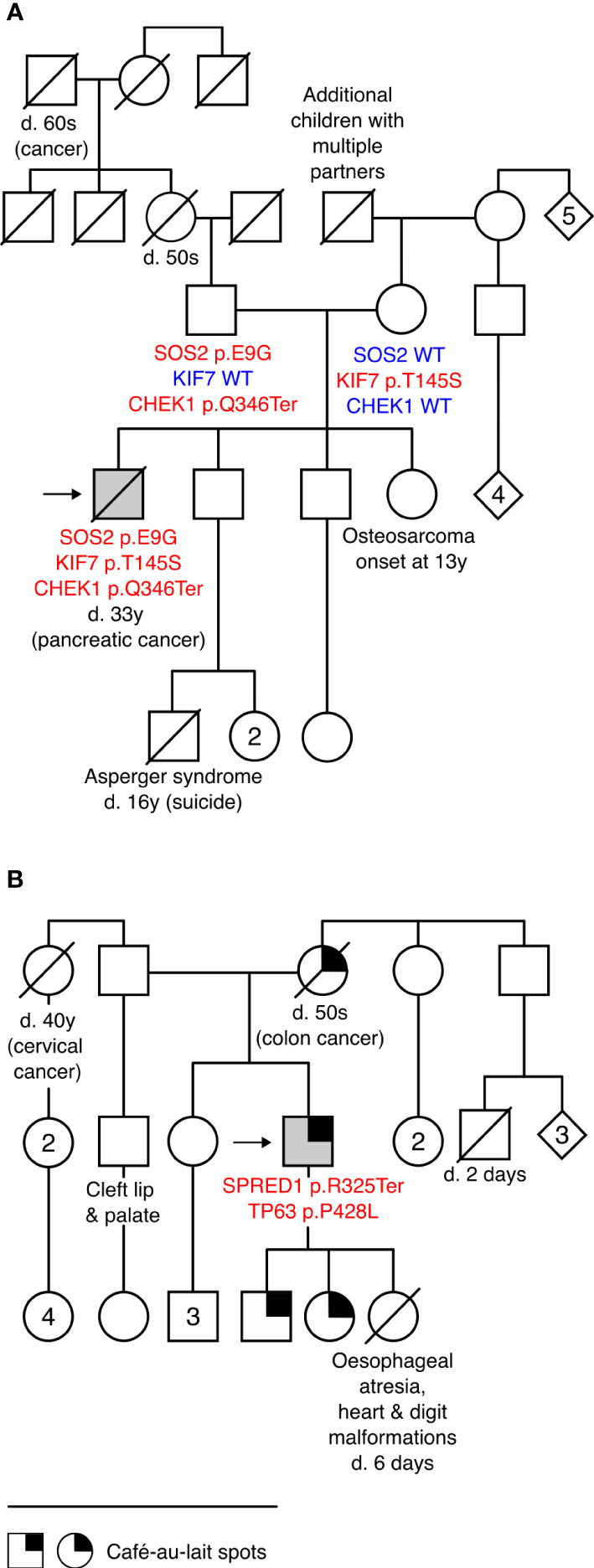
Patients with more than one disease-causing variant and their pedigrees. **(A)** Pedigree of Case 6 carrying a novel likely pathogenic (LP) variant in Noonan syndrome gene *SOS2* c.26A>G (p.E9G) and incidental findings *KIF7* c.434A>C (p.Y145S; LP) and *CHEK1* c.1036C>T (p.Q346Ter; LP) that are likely co-contributors to the patient’s complex phenotype and comorbidities. **(B)** Pedigree of Case 9 carrying *SPRED1* c.973C>T (p.R325Ter; pathogenic) and an incidental finding *TP63* c.1283C>T (p.P428L; LP). *Café-au-lait* spots, characteristic of Legius syndrome, were reported in three generations. *TP63* is linked to several congenital conditions, including hydronephrosis, urethral stenosis (21), and orofacial clefts (22), observed in this family. None of the family members was available for genetic testing. Detailed clinical data on Case 6 and Case 9 are provided in **Table 2**. d., died; y, years; WT, wild type.

Two patients were identified as carriers of additional LP/P variants in genes linked to other congenital conditions as likely co-contributors to their phenotype (**Figure 2**, **Table 2**).

The carrier of the SOS2 p.E9G (Case 6) presented typical facial features of RASopathies, gonadal dysgenesis (CR, low TTV, anejaculation, and hypogonadism), and impaired mobility and cognitive and intellectual performance. He had also been diagnosed with Lennox–Gastaut syndrome, characterized by early-onset epilepsy and pancreatic cancer that are not typically observed in *SOS2*-linked NS. As SOS2 p.E9G was inherited from a fertile father (andrological data unavailable), the effect on reproductive phenotype likely has incomplete penetrance. Two uncles and a granduncle of the patient’s father were childless (unavailable for genotyping). The patient carried two incidental LP findings: maternally inherited KIF7 p.Y145S and paternally derived CHEK1 p.Q346Ter. Monoallelic variants in *KIF7* have been linked to various congenital phenotypes (27) that overlap with the features of Case 6 (cryptorchidism, scoliosis, epilepsy, DD/intellectual disability, characteristic facial features, etc.). Computational modeling of a potential combined effect of *SOS2* and *KIF7* variants supports digenic pathogenicity with a 0.83 score (99% confidence interval; **Supplementary Table 9**). As the patient had pancreatic cancer (died at 33 y) and his sister had osteogenic sarcoma at an early age (13 y, unavailable for genotyping), the CHEK1 p.Q346Ter is a likely candidate variant for cancer predisposition (28) in this family.

Case 9 presenting oligozoospermia carried SPRED1 p.R325Ter (primary finding) and TP63 p.P428L (incidental). The patient, his two children, and his mother (who died of colorectal cancer in her 50s) presented extensive *café-au-lait* macules, a common characteristic of Legius syndrome linked to *SPRED1* (29, 30). However, his congenital urethral stenosis, hydronephrosis, and vertebral defects were not fully compatible with the features of this condition. The patient’s first child had developmental malformations causing perinatal mortality at 6 days of age, and his paternal half-brother had been born with a cleft lip and palate. Disease-causing variants in *TP63* are a known cause of birth defects with incomplete penetrance, including hydronephrosis, urethral stenosis (21), and orofacial clefts (22). Statistical modeling of the joint effect of SPRED1 p.R325Ter and TP63 p.P428L supported potential digenic pathogenicity with a 0.94 score (99.9% confidence interval, **Supplementary Table 9**).

### Four of seven GEMINI cases with RASopathy-linked variants presented cryptorchidism

**Figure 3:**
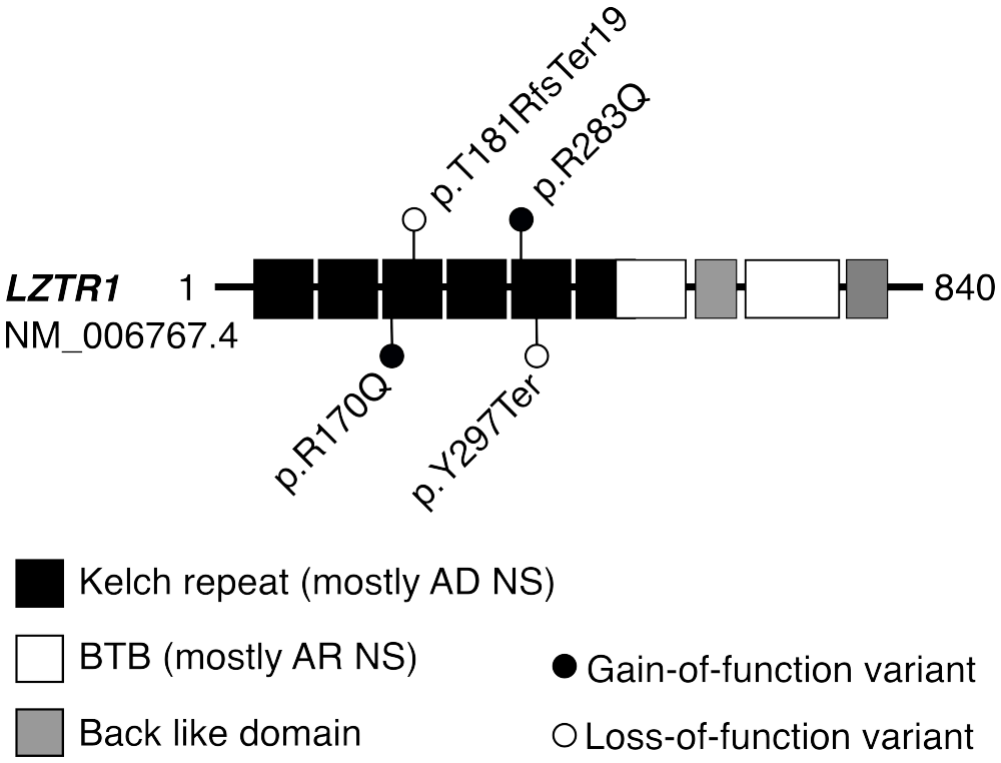
Protein domains of LZTR1 harboring variants linked to dominant or recessive forms of Noonan syndrome. Disease-causing variants in the *LZTR1* gene are linked to both autosomal dominant (AD) and recessive (AR) forms of Noonan syndrome (NS), depending on the position of the variant within the encoded protein (24). Heterozygous LP/P variants in the N-terminal Kelch repeat domain are typically linked to AD forms of *LZTR1*-linked NS, whereas AR forms are mostly caused by biallelic LP/P variants within the C-terminal BTB domain. All identified LP/P findings in the *LZTR1* gene were heterozygous, located in the Kelch repeat domain, and previously reported in the ClinVar database as LP/P. Previously, the detected missense substitutions p.R283Q and p.R170Q have been linked with AD form of NS (25) or schwannomatosis (24), respectively. *BTB*, broad complex, tram track, and bric-a-brac domain.

The GEMINI cohort was used for validation of undiagnosed RASopathy cases among men with unexplained infertility. Six SPGF subjects and one fertile subject carried LP/P findings in *PTPN11* (three), *LZTR1* (three), and *MRAS* (one). Three of these variants have been classified in the ClinVar database as pathogenic, including LZTR1 p.T181RfsTer19 identified in the current study in a normozoospermic man (**Figure 3**). Except for hormonal parameters, no further health data were available for this subject.

No statistically significant difference was detected in the prevalence of RASopathy-linked findings in the GEMINI patients (seven of 1,416; 0.5%) compared to fertile men (one of 317; 0.3%). However, four of six SPGF cases with findings had a medical history of CR (**Table 4**). These cases represent a substantial fraction of the minor group of GEMINI patients with undescended testicles (four of 31, 13%; **Supplementary Table 6**). This observation was consistent with the ESTAND data showing significant enrichment of RASopathy-linked variants in the patient subgroup “SPGF with CR” (**Table 3**).

**Table 4:** RASopathy-linked heterozygous variants identified in men recruited in the USA, Canada, and Australian centers of the GEMINI cohort.

Case(age)^a^	Variant data^b^	Clinical data^c^	Center^d^
11 (25 y)	*LZTR1* c.891T>A p.Y297Ter (P) # rs145955180 MAF 5.1 × 10^−6^	H = 180 cm; W = 154 kg; BMI = 47.5 Total sperm count per ejaculate 0.41 × 10^6^ Medical history of testicular (mal)descent unknown Multiple sclerosis (diagnosis at 26 y)	1
12 (26 y)	*LZTR1* c.509G>A p.R170Q (LP) # rs781431741 MAF 1.9 × 10^−5^	CR (left); TV 8 + 15 = 23 mL FSH 10.6 IU/L NOA; histology: SCOS Inguinal hernia; asthma and food allergies	2
13 (n.a.)	*LZTR1* c.540del p.T181RfsTer19 (P) # rs1449793938 MAF 3.9 × 10^−6^	FSH 13.8 IU/L; LH 4.8 IU/L; T 22.5 nmol/L Total sperm count per ejaculate 373.7 × 10^6^ Medical history of testicular (mal)descent unknown No other health data available	2
14 (40 y)	*PTPN11* c.362A>C p.E121A (LP) Novel variant	H = 175 cm; W = 152 kg; BMI = 49.6 TV 8 + 8 = 16 mL FSH 8.5 IU/L; LH 10.5 IU/L; T 5.5 nmol/L NOA	3
15 (29 y)	*PTPN11* c.172A>G p.N58D (P) # rs397507505 MAF n.a.	CR (bil); TV 0 + 8 = 8 mL FSH 56 IU/L; LH 15 IU/L; T 18.5 nmol/L NOA Suspected Noonan syndrome	2
16 (39 y)	*PTPN11* c.518G>T p.R173L (LP) rs369155025 MAF 1.6 × 10^−5^	H = 188 cm; W = 101 kg; BMI = 28.5 CR; TV 15 + 15 = 30 mL FSH 16 IU/L; LH 5.4 IU/L; T 15 nmol/L Total sperm count per ejaculate 0.1 × 10^6^ Asthma, frequent infections	3
17 (37 y)	*MRAS* c.359C>T p.P120L (LP) Novel variant	CR (uni); TV 3.3 + 4.9 = 8.2 mL FSH 7.8 IU/L; LH n.a.; T 30.6–34.5 nmol/L NOA; Histology: SCOS Decreased virilization and mild gynecomastia; mitral valve prolapse with preserved ejection fraction (75%)	4

Variant pathogenicity assessment (16) details are provided in **Supplementary Table 7**, and Sanger sequencing confirmation is in **Supplementary Figure 3**.

bil, bilateral; BMI, body mass index; CR, cryptorchidism; FSH, follicle-stimulating hormone; GEMINI, Genetics of Male Infertility Initiative; H, height; LH, luteinizing hormone; LP, likely pathogenic; n.a., not available; NOA, non-obstructive azoospermia; P, pathogenic; SCOS, Sertoli cell-only syndrome; TV, testis volume (left + right = total); uni, unilateral; W, weight; y, years.

a Age at the recruitment.

b Gene, cDNA and protein position, rs number, minor allele frequency (MAF; gnomAD v.2.1.1); LP/P variants reported in ClinVar (#).

c Variable retrospective medical data collected at the recruitment and available for the study.

d GEMINI centers for patient recruitment: 1, University of Utah, Salt Lake City, UT, USA; 2, Monash University, Victoria, Australia; 3, University of Toronto, Ontario, Canada; 4, Weill Cornell Medical College, New York, USA.

Notably, according to the documented medical records at the recruitment, Noonan syndrome had been previously suspected (but not confirmed by genetic testing) for the GEMINI patient carrying an established NS-linked variant PTPN11 p.N58D. The subject with MRAS p.P120L presented congenital cardiac complications, and the subject with LZTR1 p.Y297Ter presented multiple sclerosis. Both PTPN11 p.R173L and LZTR1 p.R170Q carriers had been diagnosed with asthma.

### Testicular expression profile of RASopathy-linked genes

Testicular biopsy for immunohistochemical staining was available for Case 1 carrying the PTPN11 p.N308D variant and a control subject with obstructive azoospermia but histologically active spermatogenesis (**Figure 1D**). In both subjects, the strongest staining intensity for PTPN11 was detected in the cytoplasm of primary spermatocytes, early round spermatids, and Leydig cells. Consistent with the abnormal testicular phenotype in Case 1 (**Figure 1C**, **Supplementary Table 8**), weaker PTPN11 expression was observed in spermatogenic compared to Leydig cells.

All Ras/MAPK pathway genes with LP/P findings showed considerable mRNA expression in both spermatogenic and testicular somatic cells (**Figure 1E**). Most of these genes had the highest expression in spermatocytes, except for *MRAS* and *MAP2K1* with elevated transcript levels in different stages of spermatogonia. High transcript levels of *PTPN11* and *SOS2* were also observed in Leydig and Sertoli cells, respectively. *MAP2K1* and *SPRED1* showed additionally increased expression in testicular immune cells, M1/M2 macrophages, and lymphoid T cells. Consistent with their immunomodulatory role, the respective affected patients presented dermatological conditions.

## Discussion

This study showed that men with low sperm count include a considerable number of subjects with undiagnosed RASopathies. In the ESTAND cohort, disease-causing variants in seven Ras/MAPK pathway genes were identified in nine of 521 analyzed SPGF patients (1.7%) and one subfertile normozoospermic man (**Figure 1**). A significant, ~13-fold enrichment of carriers was identified in the “SPGF with CR” compared to the CTRL subgroup (6/155, 3.9% *vs.* 1/323, 0.3%; *p* = 5.5 × 10^−3^; **Tables 2**, **3**). Of note, the incidence of RASopathies in the general population has been estimated to be 1 in 1,000 (1, 2).

Together with the findings in the GEMINI validation cohort, undiagnosed RASopathies were detected in total for 17 subjects, 15 SPGF patients, and two fertile men (**Tables 2**, **4**). The study data strongly supported the contention that the Ras/MAPK pathway genes represent a novel etiology of syndromic SPGF, frequently accompanied by cryptorchidism (10 of 15 infertility patients with findings).

Consistent with syndromic SPGF, all affected Estonian men presented congenital genitourinary anomalies, skeletal and joint conditions, and other clinical characteristics linked to RASopathies (**Figure 1B**, **Table 2**). Importantly, rare forms of malignancies (seminoma, schwannomatosis, and pancreatic cancer) were reported in half of these cases. The observed broad phenotypic expressivity and variable penetrance of identified variants was consistent with previous studies (1–4, 26, 31–34). Recently, it has been suggested that the clinical phenotype of RASopathies may also be modulated by digenic effects (35). This study identified two ESTAND patients with more than one disease-causing variant potentially contributing to the phenotype.

The study detected heterozygous LP/P variants in eight of 22 RASopathy-linked genes. More than one finding was detected in the *PTPN11* (five) and *SOS1* (three) genes. *PTPN11* and *SOS1* are the most affected NS genes, accounting for ~50% and ~10%–15% of patients, respectively (31, 32). Surprisingly, three patients carried frequently observed variants of PTPN11-linked NS (**Tables 2**, **4**). They presented concordant andrological phenotypes: CR and NOA. Notably, the ESTAND patients with PTPN11 p.N308D and p.M504V had all major RASopathy-linked clinical conditions (10, 11), and the GEMINI case carrying PTPN11 p.N58D had been suspected with NS during his medical assessment. All findings in the *SOS1* gene were missense variants, typical of SOS1-linked NS (26). Both SPGF patients with novel *SOS1* variants had CR and cryptozoospermia (**Table 2**). SOS1 p.Y136H carrier also presented dermatological conditions, frequently observed in NS patients (26). The patient with SOS1 p.Q214H had been diagnosed with testicular cancer. It has been shown that patients with *SOS1* heterozygous variants may have a higher risk for solid tumors (26).

Four LP/P variants in the *LZTR1* gene were identified in three SPGF subjects and one fertile subject (**Figure 3**). The cases with previously reported dominant missense substitutions LZTR1 p.R170Q (24) (GEMINI) and p.R283Q (25) (ESTAND) presented concordant andrological phenotypes: unilateral CR, NOA, and SCOS. The former patient was also suffering from schwannomatosis, characteristic of *LZTR1*-linked NS (34). However, no ultimate conclusions could be drawn for the phenotypic effect of heterozygous loss-of-function (LoF) variants in *LZTR1*, detected in one cryptozoospermia subject and one normozoospermia subject in the GEMINI cohort. Their data on testicular (mal)descent and general health conditions were unavailable, except for multiple sclerosis diagnosed as the carrier of LZTR1 p.Y297Ter. In the literature, *LZTR1* LoF variants have been reported in patients with schwannomatosis, glioblastoma, and autosomal recessive NS (36, 37).

*NF1* is one of the largest genes in the human genome with high genetic heterogeneity (38). The ESTAND patient with NF1 finding did not present neurofibromas and skin conditions typical of neurofibromatosis 1. However, he had been diagnosed with several orthopedic, ocular, and nervous system disorders and lymphedema, which are frequently reported in RASopathy patients (39). Rare *MRAS*-linked RASopathies are typically linked to congenital heart diseases (40). The GEMINI carrier of MRAS p.P120L presented CR and NOA (SCOS) and had been also diagnosed with mitral valve prolapse.

The gathered data suggested that on some occasions, congenital defects in the Ras/MAPK pathway may primarily manifest as a testicular phenotype. Others have also reported pathogenic variants in NS genes *SOS1*, *NRAS*, and *BRAF* in boys with isolated CR without typical characteristics of NS (41). The Ras/MAPK pathway genes with findings are highly expressed in spermatogenic and testicular somatic cells with likely contributions to testis development and function (**Figures 1D, E**). In men with NS, dysfunction of Sertoli and Leydig cells has been reported, reflected by altered reproductive hormone levels (42, 43).

Referring to the vital role of the Ras/MAPK pathway in mammalian development, homozygous knockout (KO) mouse models of most RASopathy genes have been reported to suffer embryonic or fetal lethality and various placental defects (44). However, heterozygous KO mouse models do not present male infertility. Exceptions are *Ptpn11* KO mice for whom azoospermia, spermatogenic arrest, and seminiferous tubule degeneration have been reported (45), matching the testicular histopathology observed in Case 1 (**Figure 1C**, **Supplementary Table 8**). The irrelevance of KO mouse models to the RASopathy phenotype is explained by gain-of-function changes present on most occasions (46). Also, in this study, most of the identified variants were missense substitutions. The identified truncating variant SPRED1 p.R325Ter (Case 9) is linked to Legius syndrome, a rare RASopathy caused by inactivating mutations.

*De novo* variants in the Ras/MAPK pathway genes have been shown to be under positive selection, leading to clonal expansion in the germline of aging men (47). Unfortunately, parental DNA was unavailable in most cases of this study due to the sensitive and personal nature of infertility diagnosis. As CR is the most prevalent male birth defect (48), a fraction of cases likely arises due to *de novo* mutations in the Ras/MAPK pathway genes during spermatogenesis, representing a novel potential etiology behind sporadic CR cases.

## Conclusions

This study revealed that congenital defects in the Ras/MAPK pathway genes represent a novel genetic etiology of SPGF, most likely due to affected testicular development and function. In perspective, special attention must be paid to screening RASopathy-linked variants in unexplained SPGF cases with a medical history of CR to offer optimal and timely management and counseling for reproductive and general health. Given the relationship between RASopathies and other conditions, infertile men found to have this molecular diagnosis should be evaluated for congenital genitourinary anomalies, skeletal and joint conditions, and other RASopathy-linked health concerns, including specific rare malignancies associated with this diagnosis.

## Data availability statement

All relevant details of genotype-phenotype data for this study are included in the manuscript and the supplementary files. The presented data are deposited in the NCBI ClinVar database (https://www.ncbi.nlm.nih.gov/clinvar/), accession numbers: SCV004231722 - SCV004231724, SCV004231705 - SCV004231720 and SCV002525862.1.

## Ethics statement

The studies involving humans were approved by Ethics Review Committee of Human Research of the University of Tartu, Estonia; IRB of University of Utah, USA; IRB of Weill Cornell Medical College, New York, USA; IRB of Mount Sinai Hospital, University of Toronto, Toronto, Canada; human ethics committees of Monash Surgical Day Hospital, Monash Medical Centre and Monash University, Australia. The studies were conducted in accordance with the local legislation and institutional requirements. The participants provided their written informed consent to participate in this study.

## Author contributions

A-GJ: Formal analysis, Investigation, Writing – original draft, Writing – review & editing, Validation, Visualization. KP: Investigation, Methodology, Resources, Writing – review & editing. AD: Data curation, Formal analysis, Methodology, Writing – review & editing. ET: Investigation, Methodology, Resources, Writing – review & editing, Visualization. AV: Formal analysis, Validation, Writing – review & editing. KL: Formal analysis, Validation, Writing – review & editing. EM: Formal analysis, Software, Visualization, Writing – review & editing. STj: Writing – review & editing, Investigation. GB: Writing – review & editing, Formal analysis, Validation. VK: Writing – review & editing, Investigation, Methodology. HC-M: Writing – review & editing, Data curation. AR-E: Writing – review & editing, Investigation, Validation. LP: Writing – review & editing, Formal analysis, Validation. LN: Writing – review & editing, Data curation. OP: Writing – review & editing, Investigation. VV: Writing – review & editing, Investigation. MS: Writing – review & editing, Investigation. NV: Writing – review & editing, Methodology. SP: Writing – review & editing, Methodology. RIM: Writing – review & editing, Investigation, Funding acquisition, Resources. KAJ: Writing – review & editing, Investigation, Resources. PNS: Writing – review & editing, Investigation, Resources. STe: Writing – review & editing, Investigation. PK: Writing – review & editing, Investigation. KV-C: Writing – review & editing, Visualization. MKO'B: Funding acquisition, Resources, Writing – review & editing. KIA: Data curation, Funding acquisition, Writing – review & editing. TL: Funding acquisition, Methodology, Writing – review & editing. DFC: Data curation, Funding acquisition, Writing – review & editing. LK: Formal analysis, Investigation, Methodology, Writing – review & editing. MP: Conceptualization, Data curation, Funding acquisition, Investigation, Methodology, Project administration, Resources, Supervision, Writing – review & editing. ML: Conceptualization, Data curation, Formal analysis, Funding acquisition, Investigation, Methodology, Project administration, Resources, Supervision, Writing – original draft, Writing – review & editing.
