# Supplementary material for: Undiagnosed RASopathies in infertile men
Source: Front Endocrinol (Lausanne). 2024 Apr 9;15:1312357. doi: 10.3389/fendo.2024.1312357 (PMC11035881; doi:10.3389/fendo.2024.1312357)
Supplement: Supplementary file 1 [file DataSheet_1.pdf]

# List of Supplementary Materials

**Supplementary Methods**

Study subjects and clinical data 3

Whole-exome sequencing (WES) and variant calling 4

Filtering and annotation of variants 6

Detection of disease-causing variants in Ras/MAPK pathway genes 6

Assessment of potential digenic effects 7

Variant validation by Sanger sequencing 8

Extended clinical and family data of ESTAND patients with genetic findings 8

Testicular histopathology and immunochemistry 8

Single-cell RNA-Seq dataset of testicular cells 9

Assessment of Rasopathy-linked variants in the GEMINI cohort 9

Statistical tests for differences in parameter distributions 10

**List of online platforms and tools** 11

**Supplementary Figures**

**Supplementary Figure 1.** Sanger sequencing of heterozygous *PTPN11*, *LZTR1*, *SOS1*, *NF1*, and *MAP2K1* variants identified in the ESTAND cohort. 12

**Supplementary Figure 2.** Sanger sequencing of *SPRED1* and *SOS2* variants identified in the ESTAND cohort, and incidental findings in Case 6 and Case 9. 13-14

**Supplementary Figure 3.** Sanger sequencing of heterozygous *LZTR1*, *PTPN11*, and *MRAS* variants identified in the GEMINI cohort. 15

**Supplementary Tables**

**Supplementary Table 1.** Flags and plugins in implementing Ensembl VEP v105.0. 16-17

**Supplementary Table 2.** List of analyzed RASopathy-linked genes. 18-19

**Supplementary Table 3**. Variant pre-filtering criteria after annotation with Ensembl VEP. 20

**Supplementary Table 4.** PCR and Sanger sequencing primers. 21-22

**Supplementary Table 5.** Assessment of variant pathogenicity in the ESTAND cohort. 23-24

**Supplementary Table 6.** Clinical parameters of the GEMINI cohort subgroups. 25

**Supplementary Table 7.** Assessment of variant pathogenicity in the GEMINI cohort. 26

**Supplementary Table 8.** Testicular histopathology analysis of Case 1. 27

**Supplementary Table 9.** Statistical modeling of digenic pathogenicity of the RASopathy-linked variant and incidental finding in another developmental gene in Cases 6 and 9. 28

**References to Supplementary materials** 29

## SUPPLEMENTARY METHODS

## STUDY SUBJECTS AND CLINICAL DATA

**Ethics statement.** The study was approved by the Ethics Review Committee of Human Research of the University of Tartu, Estonia (permissions no. 74/54 and 118/69 with last amendment 288/M-13; no. 221/T-6, 286/M-18). Research in the involved GEnetics of Male INfertility Initiative (GEMINI) study centers has been approved by IRB_00063950 (IRB of University of Utah, USA); 16030459 and 0102004794 (IRB of Weill Cornell Medical College, New York, USA); 15-0147-E (IRB of Mount Sinai Hospital, University of Toronto, Toronto, Canada); and human ethics committees of Monash Surgical Day Hospital, Monash Medical Centre and Monash University, Australia.

Written informed consent for evaluating and using their clinical data for scientific purposes was obtained from each patient before recruitment. The study was carried out in compliance with the Helsinki Declaration.

**Recruitment and andrological phenotyping of the ESTtonian ANDrology (ESTAND) cohort.** All study participants were recruited to the ESTAND cohort[1], and the material was collected at the Andrology Clinic of Tartu University Hospital (AC-TUH), Estonia. AC-TUH is an accredited member of the European Academy of Andrology (EAA), and all andrologists have received training in standardized clinical assessment in the EAA centers. AC-TUH represents the primary and referral center, managing >90% of all male infertility cases in the country. All 844 men analyzed in the current study had undergone identical routine andrological workup[1] by andrology specialists using the established clinical pipeline and standard protocols at AC-TUH. The couple's infertility was diagnosed as a failure to conceive for a period of ≥12 months. All participants were of white European ancestry and living in Estonia.

**Physical examination** included the assessment of genital phenotype and testicular size with an orchidometer (birch wood, Pharmacia & Upjohn, Denmark) in a standing position. Details for the total testis volume (sum of left and right testicles), the position of the testicles in the scrotum, pathologies of the genital ducts, penis, urethra, and presence and grade of varicocele were registered for each patient. Reduced bitesticular volume was defined as <30 mL.

Patients with at least one testicle missing in the scrotum at the recruitment or medical history of orchidopexy were diagnosed with congenital cryptorchidism (CR).

**Sperm analyses** were conducted according to the World Health Organization (WHO) recommendations[2] on samples acquired by patient masturbation. The semen samples were incubated at 37°C for 30-40 minutes for liquefaction. Then, the volume was estimated by weight, subtracting the collection tube weight and assuming 1 g = 1 mL. The concentration of spermatozoa was estimated on diluted samples (with 0.6 mol/L $NaHCO_3$%, 0.4% (v/v) formaldehyde in distilled water) using the improved Neubauer hemocytometers. Spermatogenic failure (SPGF, total sperm counts 0-39×$10^6$ per ejaculate) was defined according to the WHO guidelines[2]. Reference ranges for sperm analysis are provided in **Table 1**. Non-obstructive azoospermia (NOA) refers to a complete lack of sperm in the ejaculate due to primary SPGF. Oligozoospermia is defined as >0 and ≤39×$10^6$ sperm per ejaculate. Normozoospermia refers to total sperm counts >39×$10^6$ per ejaculate. SPGF due to obstruction was assessed and excluded during the andrological workup, including extended assessment of the carriership of *CFTR* biallelic variants from the generated exome sequencing data.

**Blood samples** for hormonal analysis were taken between 8 a.m. and 10:30 a.m. on the same day the semen sample was produced. Blood was centrifuged, and serum was used to determine testosterone, follicle-stimulating hormone (FSH), and luteinizing hormone (LH) levels by electrochemiluminescence immunoassay (ECLIA) method (Cobas e 601, Roche Diagnostics). All analyses were carried out at the United Laboratories, Tartu University Hospital, and the reference ranges are provided in **Table 1**.

**Formation of the ESTAND study group for whole-exome sequencing (WES)**. The study group for WES included 844 ESTAND participants: 521 patients with idiopathic SPGF (median age at recruitment 34 years) and 323 normozoospermic partners of pregnant women as a control group (CTRL; 31 years; median sperm counts $303.1\times10^6$ per ejaculate; **Table 1**). SPGF was defined as 0-39 million sperm per ejaculate[2]. SPGF patients were prioritized to WES based on their severe unexplained SPGF suggesting a possible genetic cause (e.g., extremely low sperm counts, abnormal hormonal parameters, genitourinary conditions). Patients with at least one testicle missing in the scrotum at the recruitment or medical history of orchidopexy formed the 'SPGF with CR' subgroup (n=155), whereas the rest were defined as 'SPGF only' (n=366). The two subgroups did not differ in andrological parameters, except for lower total testis volume (TTV) in CR cases (**Table 1**).

The CTRL subjects had been recruited during an ongoing pregnancy of the female partner[1,3]. Sperm quality parameters (motility and morphology) and time to pregnancy were not considered as exclusion/inclusion criteria for the control group. The latter information was not available for most patients. Given this limitation, the CTRL group may include men with other forms of male (sub)infertility apart from quantitative SPGF which was the focus of the formation of this study group.

The study group included five patients from previously published family studies: (i) one pair of brothers with the same genetic finding[4]; and (ii) one pair of first cousins and additionally their joint first cousin once removed with variable genetic findings[5]. The rest of the study group represented 516 unrelated singleton SPGF cases and 323 CTRL men.

All subjects (n=844) were tested for cytogenetic abnormalities. According to the international guidelines[6], patients who presented spermatozoa concentrations of ≤5 million/ml (n=352) were analyzed for Y-chromosome microdeletions[1]. None of the subjects included into the study cohort presented gross chromosomal abnormalities or Y-chromosomal microdeletions.

**Exclusion criteria from the study group for WES.** Patients diagnosed with known genetic and non-genetic causal factors for male infertility[1,2], such as secondary hypogonadism, seminal tract obstruction, cytogenetic abnormalities, Y-chromosome microdeletions, sexual dysfunction, androgen abuse, severe traumas and operations in the genital area or chemo- or radiotherapy were excluded from this study.

## WHOLE-EXOME SEQUENCING (WES) AND VARIANT CALLING

**WES data generation and processing.** WES was performed using genomic DNA extracted from blood samples. NGS library preparation, sequencing, primary sequence analysis, and variant calling were performed in three sequencing centers: 447 DNAs were sequenced at the Next Generation Sequencing Service laboratory of the Institute for Molecular Medicine Finland (FIMM), Helsinki, Finland (funding Estonian Research Council project PRG1021, PI: Maris Laan); 82 DNAs were sequenced at the McDonnell Genome Institute of Washington

University in St. Louis, MO, USA and 315 at the Huntsman Cancer Institute High-Throughput Genomics Core Facility at the University of Utah (funding NIH project R01HD078641, GEMINI; co-PI-s Donald F Conrad and Kenneth I Aston).

**WES pipelines at the NGS Service at the FIMM, Helsinki, Finland.** 50 ng of gDNA was processed according to the Twist Human Core Exome EF Multiplex Complete kit (Twist Bioscience, San Francisco, CA, USA) manual. 4 µL of 15 µM Adapters used for ligation were unique dual index (UDI) oligos with unique molecular barcodes (UMI) by IDT (Integrated DNA Technologies, Coralville, IA, USA). Library quantification and quality check were performed using LabChip GX Touch HT High Sensitivity assay (PerkinElmer, USA) and Qubit Broad Range DNA Assay (Thermo Fisher Scientific, Waltham, MA, USA). Libraries were pooled to 8-plex reactions according to concentration. The exome enrichment was performed using Twist Comprehensive Exome probes. The captured library pools were quantified for sequencing using KAPA Library Quantification Kit (KAPA Biosystems, Wilmington, MA, USA) and LabChip GX Touch HT High Sensitivity assay. Sequencing was performed with the Illumina NovaSeq system using S2 flow cell (Illumina, San Diego, CA, USA) and v1.5 chemistry. The read length for the paired-end run was 2×101 bp. Sequencing resulted in an average of 59 million reads per exome with an average median target coverage of 66X. Primary sequencing analysis and variant calling were performed using the Illumina DRAGEN Bio-IT Platform (v3.9 and v3.10, human genome build hg38). Only cases with normal sex-chromosomal ploidy estimations derived from the DRAGEN output were taken forward to exclude undiagnosed cases with 47,XXY or other sex chromosomal abnormalities prevalent in infertile men.

For eight subjects, WES data had been generated using the previous in-house pipeline at the FIMM NGS Service, described by[4,5]. Whole exome enrichment was undertaken with the SeqCap EZ MedExome Target Enrichment Kit (Roche NimbleGen, Madison, WI, US) and sequencing was performed on Illumina HiSeq 2500 sequencing system (San Diego, CA, USA) using paired-end 101 bp read length. Sequencing resulted in an average of 96 million reads per exome with a mean target coverage of 104X. Primary sequence analysis and variant calling were performed using the Variant Calling Pipeline (VCP3.7)[7].

**WES pipelines of the GEMINI study.** WES data generation for 82 ESTAND SPGF patients included in the GEMINI study has been recently described in detail[8]. Briefly, WES was performed at the McDonnell Genome Institute of Washington University in St. Louis, MO, USA (genome.wustl.edu) using an in-house exome targeting reagent capturing 39.1 Mb of exome and 2×150 bp paired-end sequencing on Illumina HiSeq 4000. Average exome coverage was 80X across sequenced individuals.

Most of the ESTAND CTRL group (n=315) was sequenced by the Huntsman Cancer Institute High-Throughput Genomics Core Facility at the University of Utah. Genomic DNA libraries were constructed using high molecular weight DNA using the Illumina DNA Prep with Enrichment kit (cat#20025523). PCR-amplified libraries (500 ng) were enriched for exonic regions by hybridization with the IDT xGEN Exome Research Panel v2 (cat#10005152), and libraries were sequenced using 2×150 bp paired-end sequencing on Illumina NovaSeq S4.

In the current study, all ESTAND WES datasets generated by the GEMINI pipelines (total n=397) were jointly reanalyzed with an additional 1,855 WES datasets (of other populations) as part of the GEMINI "Phase II". This reprocessing, which entailed all steps from read mapping to genotype calling, was performed at the Utah Center for Genetic Discovery, using UCGD pipeline (v2.13). Raw sequencing reads were processed and aligned to GRCh38

assembly in an alternate contig aware manner using bwa-mem v0.7.1776[9] and duplicate read marking with SAMBLASTER (v0.1.26)[10]. Joint genotype calling of all samples was done using Genome Analysis Toolkit (GATK)[11].

## FILTERING AND ANNOTATION OF VARIANTS

**Processing of VCF files and variant annotation using Variant Effect Predictor.** Exome sequencing data generated in FIMM (Helsinki, Finland) were delivered as individual sample variant call format (VCF) files. The data generated in the GEMINI project (St. Louis, MO, USA) were received as a single merged VCF file containing all variants of all the processed ESTAND samples and was subsequently split into individual sample files for further bioinformatic processing. All individual sample VCF files generated in both sequencing service centers were filtered for quality using identical parameters. Variants with low depth of coverage (DP <10) and low genotype quality (GQ <20) were excluded. Individual samples were further processed by filtering out heterozygous variants in the non-PAR regions of chromosomes X (GRCh38 assembly, chrX:1-10000; chrX:2781480-155701382; chrX:156030896-156040895) and chromosome Y (chrY:1-10000; chrY:2781480-56887902; chrY:57217416-57227415).

All individual filtered VCF files were merged into a single VCF file and then segmented into individual chromosome files (n=24: chr1-22, X, and Y) for variant annotation. Merging, filtering, and splitting of VCF files was performed with bcftools v.1.14[12]. The individual chromosome VCF files (n=24) were annotated with Ensembl Variant Effect Predictor (VEP) version 105[13] in the offline mode using the set of flags and plugins as listed in **Supplementary Table 1**. Variants & genotypes in 22 Ras/MAPK pathway genes (**Supplementary Table 2**) with unlikely causative link to RASopathies were excluded using custom filters applied to the annotated VEP files (**Table S3**). Only missense, frameshift, stop-gain, start-lost, and splice region/acceptor/donor variants of the canonical transcript were considered with minor allele frequency (MAF) ≤1% in gnomAD (v2.1.1) database across all subjects. The applied filters excluded known likely benign and benign variants (e.g., based on ClinVar[14] records) and variants with a low probability to be pathogenic (CADD ≤ 10, SIFT: benign, PolyPhen: tolerated). The variants that passed all the above inclusion and exclusion criteria were then considered for further analysis.

## DETECTION OF DISEASE-CAUSING VARIANTS IN Ras/MAPK PATHWAY GENES

**Formation of the candidate gene list.** *In-silico* gene panel comprised of 22 genes linked to the Ras/MAPK pathway (**Table S2**). Assembling the panel, information from different databases like Online Mendelian Inheritance in Man (OMIM, https://www.omim.org), Human Phenotype Ontology (HPO, https://hpo.jax.org/app/), and publications addressing the genetics of RASopathies was gathered and taken into consideration.

**Filtering and prioritization of variants in the RASopathy genes.** VEP output files were subjected to three stages of variant filtering – custom-designed automatic exclusion of variants with an unlikely disease-causing effect (*see above*, **Supplementary Table 3**), implementation of an advanced AI-based interpretation engine for variant pathogenicity prediction to shortlist the most likely variants with a disease-causing effect, and final manual assessment. All but two RASopathy-linked genes are considered autosomal-dominant (AD). *SPRED2* is an autosomal-recessive (AR) gene, and only biallelic likely pathogenic/pathogenic (LP/P) variants cause a phenotype; disease-causing variants in a more recently uncovered RASopathy gene *LZTR1* have been shown to affect either in AD or AR manner depending on the location within the

gene[15] (**Figure 2**). Therefore, both inheritance modes were considered in analyzing the *LZTR1* gene variants based on the location of variants within the gene.

The classification of retained variants (heterozygous in AD genes; biallelic in *SPRED2*; bi- and monoallelic in *LZTR1*) was based on the American College of Medical Genetics and Genomics (ACMG) guidelines customed to the RASopathy genes[16]. The initial interpretation of variant pathogenicity was facilitated by the free online AI-based platform Franklin by Genoox (https://franklin.genoox.com). This platform uses all the available sources of information for variant pathogenicity prediction. It provides additional information regarding the variant and gene, such as linked phenotypes, Pubmed records, and ClinVar data[14]. It represents the largest real-time genomic database supported by various advanced genomic tools and applications[17]. All variants predicted as LP or P, or variants of uncertain significance (VUS) by the Franklin machine-learning tool were included in the final manual assessment performed by two researchers in parallel. Variants predicted to be likely benign (LB) or benign (B) by Franklin were excluded from the manual inspection.

Pathogenicity of retained variants was assessed manually according to the RASopathy-specific ACMG guidelines[16] while also considering recent literature and database records, and data collected during this study, e.g., clinical and pedigree data gathered from medical records and patient interviews (*see below*). In addition, all retained variants passed a visual inspection of the quality of sequencing reads using the Integrative Genomics Viewer (IGV) software[18]. Low-confidence variant calls were discarded.

## ASSESSMENT OF POTENTIAL DIGENIC EFFECTS

**Analysis of the whole WES dataset in patients with disease-causing variants identified in the RASopathy genes.** To assess whether the identified LP/P variant in the RASopathy-linked gene was a true monogenic cause explaining the patient's phenotype, all 10 subjects with identified disease-causing variants underwent extended analysis across all genes in the WES dataset. Variant filtering followed the established criteria (**Supplementary Table 3**), except for a more stringent CADD score (>20) to prioritize high-impact variants with a robust interpretation of the effect.

**Prediction of digenic variant combinations using the ORVAL platform.** Digenic variant combinations were tested using the bioinformatics platform ORVAL (Oligogenic Resource for Variant AnaLysis) (v3.0.0, GRCh38 assembly) (https://orval.ibsquare.be). The platform uses innovative machine learning methods for combinatorial variant pathogenicity prediction, exploring pathogenic gene and protein interaction networks, and ranking pathogenic gene pairs[19]. To make predictions, the platform uses VarCoPP2.0 (Variant Combination Pathogenicity Predictor 2.0)[20]. VarCoPP2.0 is a machine learning method trained on Oligogenic Diseases Database (OLIDA)[21] against a large subset of variant data (consisting of only exonic and splicing variants of MAF <3.5% from only protein-coding genes) of control individuals from the 1000 Genomes Project (1KGP). The method uses Balanced Random Forest predictor consisting of 400 decision trees, generating a score (0-1) to predict whether the bi-locus variant combinations are disease-causing or neutral. The variant combinations with VarCoPP2.0 scores below 0.5 are predicted to be neutral and ≥0.5 to be disease-causing. Variant combinations with a score of 0.647–0.849 are predicted to be disease-causing with 99% confidence, and combinations with a score ≥0.850 are predicted to be disease-causing with 99.9% confidence.

## VARIANT VALIDATION BY SANGER SEQUENCING

All reported variants in this study were validated with Sanger sequencing. Primers were designed using Primer3 (v4.1.0)[22], Primer-BLAST[23], GenomeTester (v1.3)[24], and Benchling platform (https://www.benchling.com). PCR protocol was optimized for each primer pair, and products were run on a 1.0% or 1.5% 0.5x TBE gel for 45 minutes. Sanger sequencing was performed at the Core Facility of Genomics (Tartu, Estonia) (https://genomics.ut.ee/en/genomics-core-facility). Acquired sequence data (Geospiza, Inc., Seattle, WA, USA; http://www.geospiza.com) was analyzed using FinchTV (v1.4) and 4Peaks (v1.8). PCR and sequencing primers are presented in **Supplementary Table 4**.

The validation of all variants by Sanger sequencing is shown in **Supplementary Figures 1-3**. A previously published ESTAND cohort case carrying *SOS1* c.406T>C p.Tyr136His was included in the study for the completeness of the information about the cohort as a whole. This variant has been validated with Sanger sequencing in the original study[5].

## EXTENDED CLINICAL AND FAMILY DATA OF ESTAND PATIENTS WITH GENETIC FINDINGS

All 10 ESTAND subjects with LP/P variants in the RASopathy genes (**Table 2, Supplementary Table 5**) were invited for individual feedback and follow-up assessment. Eight of them accepted the offer. The patient's physical and facial characteristics, health conditions, and family health history were gathered and considered during the sessions. The interviews were conducted at the ambulatory facilities of AC-TUH. The principal investigator carried out each session, supported by the managing andrologist. One to two research assistants documented the interviews.

All subjects who consented to feedback and follow-up assessment in the presence of the managing andrologist underwent observational phenotyping and gathering of the family health history, carried out in parallel by two or more researchers. Established recommendations for clinical phenotyping patients with RASopathy syndromes was followed, including common congenital conditions, health comorbidities, and characteristic facial features typical to RASopathy patients[25-26].

When consented by the patient, additional family members were invited to the cascade screening of the identified genetic variant(s). The gDNA from family members was extracted from the blood samples using PureLink™ Genomic DNA Mini Kit (Thermo Fisher Scientific Inc., Waltham, MA, USA) following the manufacturer's protocol. Sanger sequencing was applied for targeted genotyping.

## TESTICULAR HISTOPATHOLOGY AND IMMUNOCHEMISTRY

**Histological profiling of testis biopsies.** Testis biopsies were collected for 110 of 521 (21.0%) analyzed SPGF cases during their infertility workup. In the clinical setting, this invasive

procedure was undertaken only when justified for clinical decision making in infertility management.

The histopathological examination was carried out at ISO-15189 accredited dedicated laboratories at the Pathology Centre, Diagnostic Clinic of the East Tallinn Central Hospital (Tallinn, Estonia) and Pathology Department, Tartu University Hospital (Tartu, Estonia). All testis specimens were taken during open surgery and immediately fixed in the Bouin's solution for 10 h, followed by additional fixation in 10% buffered formalin for 6 h. Automated tissue processing was performed with the THERMO FISHER Excelsior tissue processor. Tissue sections of paraffin-embedded biopsies were cut in a standard rotary microtome and stained with hematoxylin-eosin method. Manual testis histology evaluation in the clinical practice was performed by trained histopathology experts of the two centers, according to the guidelines[27]. For the research purposes, slides were scanned with 3D Histec Pannoramic 250 Flash III scanner; morphology assessment and measurements were performed by an experienced pathologist on digital slides with SlideViewer 2.6 software (both instruments from 3DHISTECH Ltd., Budapest, Hungary).

**Immunohistochemical detection of the testicular expression of the PTPN11 protein.** To identify and localize the PTPN11 protein in human testicular tissue, 3 mm paraffin-embedded samples from Case 1 (PTPN11 p.N308D) and a control subject (38-year-old man with obstructive azoospermia) with histologically normal active spermatogenesis, were analyzed using immunohistochemistry. All biopsies had been collected and handled at the Pathology Centre, Diagnostic Clinic of the East Tallinn Central Hospital (Tallinn, Estonia).

The paraffin-embedded tissue sections were processed with Ventana BenchMark ULTRA, fully automated for immunostaining. The sections were deparaffinized with EZ-prep solution at 72°C. Ultra LCS solution was used to provide a barrier between aqueous solutions and air during immunostaining. Ultra-Cell Conditioning 1 was used for Heat-Induced Epitope Retrieval (HIER) at 100°C for 72 minutes. The monoclonal rabbit anti-SHP2 (Y478) antibody (LOT: GR3441702-3; Abcam, Cambridge, UK) was diluted in Antibody Diluent (LOT: 41520723, Dako, Agilent, Santa Clara, USA) in the range of 1:10. The incubation lasted for 60 minutes at 37°C. The antigen-antibody complex was visualized by using Ventana OptiView DAB IHC Detection Kit (LOT: J26787) and Ventana OptiView Amplification Kit (LOT: J07004), using the Ventana Reaction Buffer (LOT: J19142) as the washing buffer between the steps (all Roche Diagnostics GmbH, Mannheim, Germany). The slides were counterstained with Hematoxylin and Bluing Reagent, each for 8 min, followed by dehydration and coverslipping with TissueTec® Coverslipping Film (Sakura Finetek, CA, USA). Immunohistochemical slides were scanned at 400x magnification and the assessment on digital slides was performed using equipment described above.

## ASSESSMENT OF RASOPATHY-LINKED VARIANTS IN THE GEMINI COHORT

Additional assessment for RASopathy-linked variants was performed in 1,733 subjects from the international GEMINI cohort (PI: D.F. Conrad and K.I. Aston). This cohort has been mainly formed to discover novel monogenic causes of NOA among idiopathic cases recruited in andrology clinics worldwide[8]. In the validation dataset of the current study, 82 ESTAND cases included to the original GEMINI cohort[8], were left out as already included in the discovery dataset with the rest of the ESTAND subjects as described above. The validation cohort of the GEMINI subjects, 1,416 SPGF patients (1,119 NOA, 297 oligozoospermia) and 317 fertile

controls (**Supplementary Table 6**) have been exome sequenced in McDonnell Genome Institute of Washington University in St. Louis, MO, USA, and the Huntsman Cancer Institute High-Throughput Genomics Core Facility at the University of Utah laboratories. Diagnoses of idiopathic SPGF followed the guideline of EAA[6] and American Urological Association/ American Society for Reproductive Medicine (https://www.auanet.org/guidelines-and-quality/guidelines/male-infertility). All subjects were screened for Y-chromosome microdeletions, cytogenetic abnormalities, and *CFTR* pathogenic variants, to exclude patients with possible obstructive azoospermia.
Further methodological details of WES data generation and filtering are provided above and interpretation of retained variants in **Supplementary Table 7**.

## SINGLE-CELL RNA-SEQ DATASET OF TESTICULAR CELLS

**scRNA-seq dataset for the analysis of gene expression of the RASopathy-linked genes.** Gene expression levels in distinct testicular cell types were derived from the human testis single-cell RNA sequencing (scRNA-seq) dataset aggregated and available through the human infertility single-cell testis atlas (HISTA v2.9.6)[28]. The collection of these human samples, as well as the bioinformatic pipelines and computational methods employed, have been previously described[28]. In summary, the data underwent careful curation and integration through specialized machine-learning techniques to minimize technical and batch-related variability. For the specific analysis in this study, HISTA's dataset was acquired (https://github.com/eisascience/HISTA) and utilized to selectively extract the scRNA-seq data representing six adult control men. The interactive web version is accessible through https://conradlab.shinyapps.io/HISTA/ and https://github.com/eisascience/HISTA is where the code and data can be downloaded.

## STATISTICAL TESTS FOR DIFFERENCES IN PARAMETER DISTRIBUTIONS

Mann-Whitney U-test was used to assess statistical differences in the distribution of quantitative parameters and Fisher's exact test for the comparison of qualitative parameters between study subgroups. All nominal P-values were adjusted for the total number of performed tests (16 parameters x 3 comparisons = 48 tests). The adjusted significance threshold was $P < 0.00104$. All statistical analyses were performed in R software (v4.0.2).

**LIST OF ONLINE PLATFORMS AND TOOLS**

| Platform / tool | Website |
|---|---|
| Benchling | https://www.benchling.com |
| ClinVar | https://www.ncbi.nlm.nih.gov/clinvar/ |
| Combined Annotation Dependent Depletion (ver 1.6) | https://cadd.gs.washington.edu |
| Ensembl | https://www.ensembl.org/index.html |
| Franklin (ver 65.1) | https://franklin.genoox.com/clinical-db/home |
| Genome Aggregation Database (ver 2.1.1) | https://gnomad.broadinstitute.org |
| Genome Tester (ver 1.3) | https://bioinfo.ut.ee/genometester/ |
| Human Phenotype Ontology | https://hpo.jax.org/app/ |
| HISTA (ver 2.9.6) | https://conradlab.shinyapps.io/HISTA/ |
| MedlinePlus | https://medlineplus.gov |
| Mouse Genome Informatics (ver 6.22) | https://www.informatics.jax.org |
| Genetic and Rare Diseases Information Center | https://rarediseases.info.nih.gov |
| Oligogenic Resource for Variant AnaLysis (ver 3.0.0) | https://orval.ibsquare.be |
| Online Mendelian Inheritance in Man | https://www.omim.org |
| Primer3 (ver 4.1.0) | https://primer3.ut.ee |
| Primer-BLAST | https://www.ncbi.nlm.nih.gov/tools/primer-blast/ |
| SlideViewer (ver 2.6) | https://www.3dhistech.com/downloads/slideviewer-2-6/ |
| The Human Protein Atlas (ver 21.1) | https://www.proteinatlas.org |
| UCSC Genome Browser | https://genome.ucsc.edu |
| Varsome (ver 11.2) | https://varsome.com |

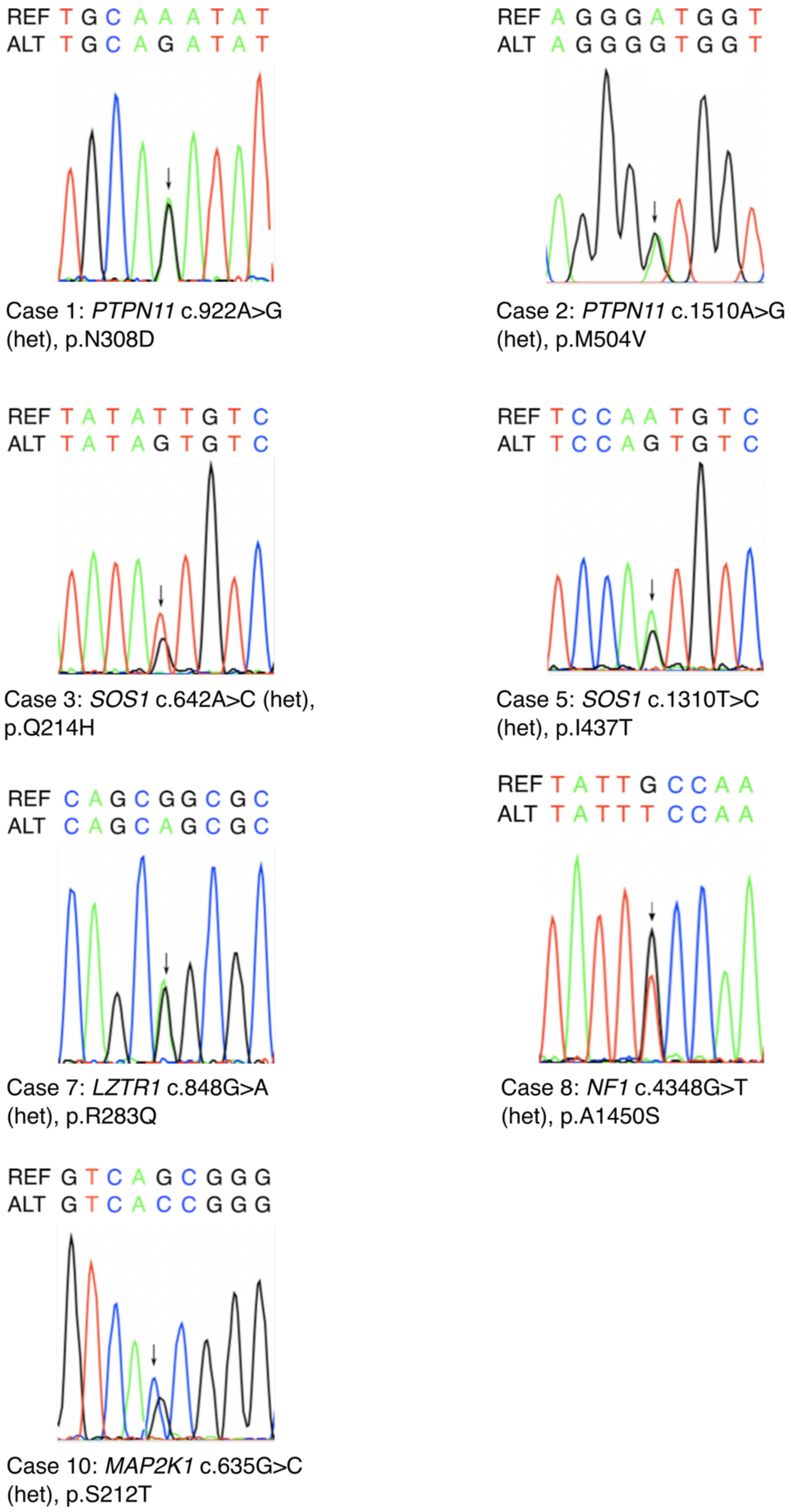


**Supplementary Figure 1. Sanger sequencing of heterozygous *PTPN11*, *LZTR1*, *SOS1*, *NF1*, and *MAP2K1* variants identified in the ESTAND cohort.** PCR and sequencing primers are provided in **Supplementary Table 4.**

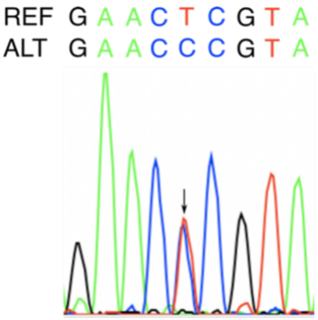


Case 6 (patient): *SOS2* c.26A>G (het), p.E9G

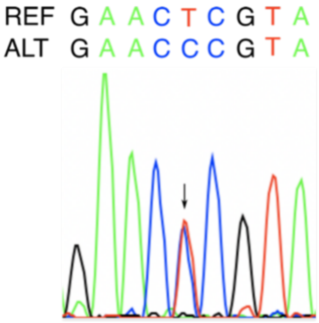


Case 6 (father): *SOS2* c.26A>G (het), p.E9G

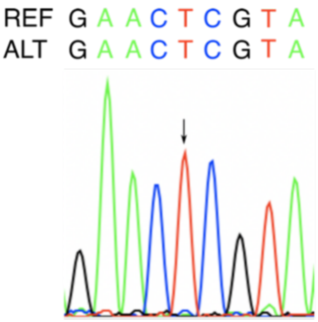


Case 6 (mother): *SOS2* c.26A (hom, WT)

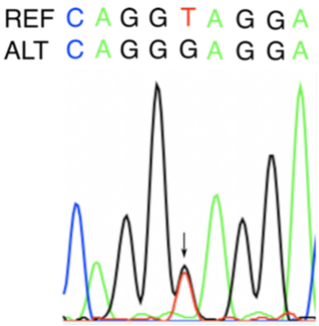


Case 6 (patient): *KIF7* c.434A>C (het), p.Y145S

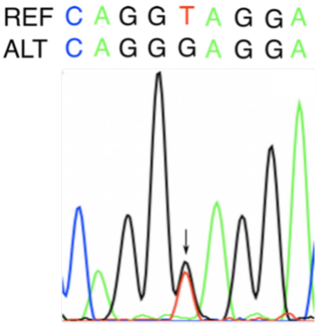


Case 6 (mother): *KIF7* c.434A>C (het), p.Y145S

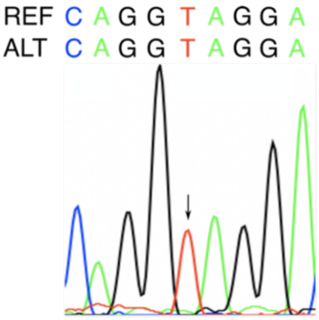


Case 6 (father): *KIF7* c.434A (hom, WT)

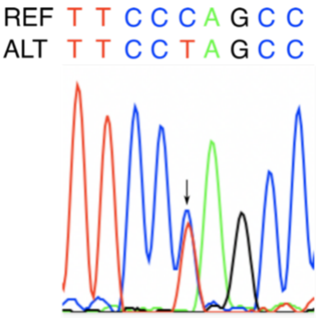


Case 6 (patient): *CHEK1* c.1036C>T (het), p.Q346Ter

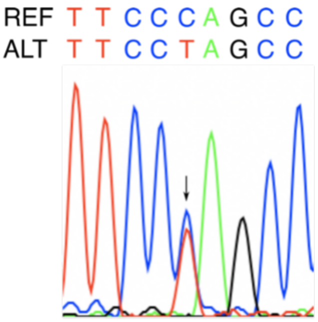


Case 6 (father): *CHEK1* c.1036C>T (het), p.Q346Ter

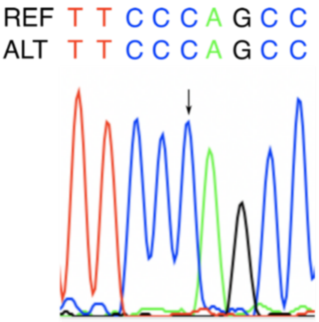


Case 6 (mother): *CHEK1* c.1036C (hom, WT)

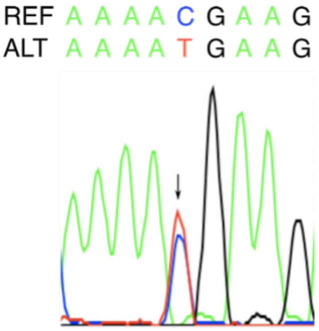


Case 9: *SPRED1* c.973C>T (het), p.R325Ter

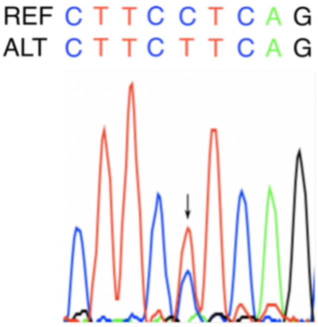


Case 9: *TP63* c.1283C>T (het), p.P428L

**Supplementary Figure 2. Sanger sequencing of *SPRED1* and *SOS2* variants identified in the ESTAND cohort, and incidental findings in respective clinical subjects Case 6 and Case 9.** PCR and sequencing primers are provided in **Supplementary Table 4**. het, heterozygous; hom, homozygous; WT, wild type

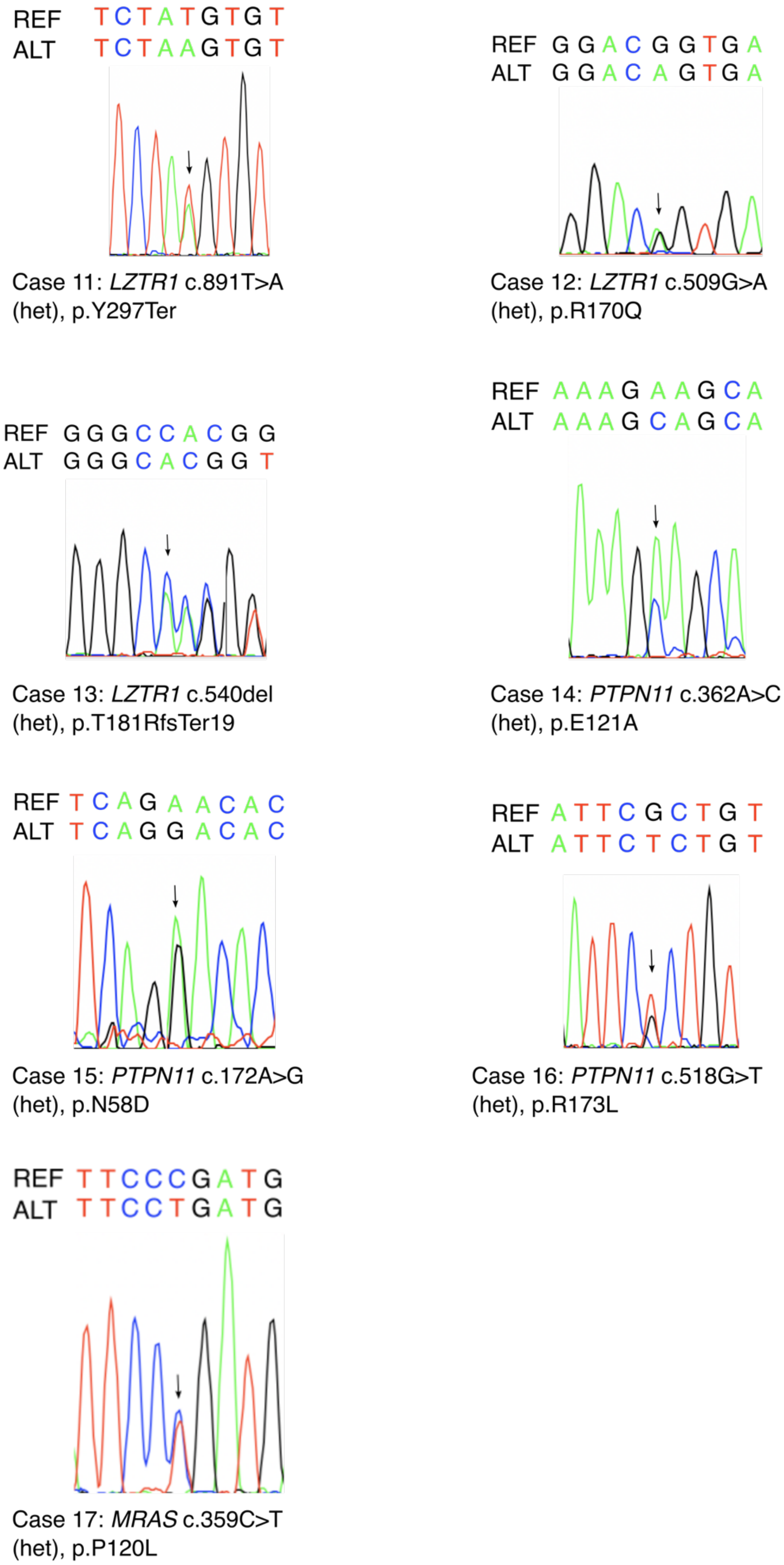


**Supplementary Figure 3. Sanger sequencing of heterozygous *LZTR1*, *PTPN11*, and *MRAS* variants identified in the GEMINI cohort.** PCR and sequencing primers are in **Supplementary Table 4**.

**Supplementary Table 1. Flags and plugins in implementing Ensembl VEP v105.0**

| Category | | Flags and plugins | Description |
|---|---|---|---|
| **Flags** | Run options | --cache | Enables the use of data from the cache. |
| | | --stats_test | Generates the stats in a plain text file. |
| | | --tab | Generates the output in a tab-delimited format. |
| | | --assembly GRCh38 | Specifies the assembly version. |
| | | --species homo_sapiens | Specifies the species of the data. |
| | Output options | --variant_class | Retrieves the Sequence Ontology variant class. |
| | | --sift b | Retrieves SIFT prediction term and score. |
| | | --polyphen b | Retrieves PolyPhen prediction term and score, using the default humVar score. |
| | | --gene_phenotype | Reports whether the overlapped gene is associated with a phenotype, disease, or trait. |
| | | --regulatory | Retrieves the feature type of RegulatoryFeature or MotifFeature by looking for overlaps with regulatory regions. |
| | | --show_ref_allele | Retrieves the reference allele. |
| | | --numbers | Retrieves the affected exon and intron. |
| | | --individual all | Retrieves all the individual IDs seperately by considering only alternate alleles present in the genotypes of the specified individual(s). |
| | Identifiers | --transcript_version | Outputs the version numbers to Ensembl transcript identifiers. |
| | | --protein | Outputs the Ensembl protein identifier. |
| | | --symbol | Outputs the gene symbol (e.g. HGNC). |
| | | --ccds | Outputs the CCDS transcript identifier. |
| | | --canonical | Indicates if the transcript is the canonical transcript for the gene by adding a flag. |
| | | --mane | Indicates if the transcript is the MANE Select or MANE Plus Clinical transcript for the gene by adding a flag. |
| | | --biotype | Outputs the biotype of the transcript or regulatory feature. |
| | | --domains | Outputs names of overlapping protein domains. |
| | | --xref_refseq | Outputs aligned RefSeq mRNA identifier for transcript. |
| | Co-located variants | --check_existing | Checks for the existence of known variants that are co-located with the input by using the default |

| | | |
|---|---|---|
| | | parameter of comparing the alleles and variants on an allele-specific basis. |
| | --max_af | Reports the highest allele frequency observed in any population from 1000 genomes, ESP or gnomAD. |
| | --var_synonyms | Reports the Pubmed IDs for publications citing the existing variant. |
| | --pubmed | Reports the known synonyms for co-located variants. |
| | --af_gnomad | Outputs the allele frequency from Genome Aggregation Database (gnomAD) exome populations. |
| | --clin_sig_allele 0 | Outputs all known clinical significance values at the given locus. |
| | --custom | Annotates the variants using the ClinVar data as custom annotation. |
| **Plugins** | --plugin dbNSFP | Retrieves the data for missense variants from a tabix-indexed dbNSFP file. |
| | --plugin CADD | Retrieves the CADD scores for variants from one or more tabix-indexed CADD data files. |
| | --plugin SpliceAI | Retrieves the pre-calculated annotations from SpliceAI (a deep neural network, developed by Illumina, Inc that predicts splice junctions from an arbitrary pre-mRNA transcript sequence) |

**Supplementary Table 2. List of analyzed RASopathy-linked genes.**

| Symbol [alias] | Gene name | Phenotype (OMIM) | MIM code | Location Inheritance |
|---|---|---|---|---|
| *BRAF [BRAF-1, BRAF1]* | B-Raf proto-oncogene, serine/ threonine kinase | Cardiofaciocutaneous syndrome; LEOPARD syndrome 3; Noonan syndrome 7 | 115150; 613707; 613706 | 7q34 AD |
| *CBL [CBL2, RNF55, c-Cbl]* | Cbl proto-oncogene | Noonan syndrome-like disorder with or without juvenile myelomonocytic leukemia | 613563 | 11q23.3 AD |
| *HRAS [HRAS1]* | HRas proto-oncogene, GTPase | Congenital myopathy with excess of muscle spindles; Costello syndrome | 218040 | 11p15.5 AD |
| *KRAS [K-Ras4B, KRAS1, KRAS2]* | KRAS proto-oncogene, GTPase | Cardiofaciocutaneous syndrome 2; Noonan syndrome 3; RAS-associated autoimmune leukoproliferative disorder | 615278; 609942; 614470 | 12p12.1 AD |
| *LZTR1 [BTBD29, LZTR-1]* | leucine zipper like transcription regulator 1 | Noonan syndrome 10; Noonan syndrome 2; Schwannomatosis-2, susceptibility to | 616564; 605275; 615670 | 22q11.21 AD, AR |
| *MAP2K1 [MAPKK1, MEK1, PRKMK1]* | mitogen-activated protein kinase kinase 1 | Cardiofaciocutaneous syndrome 3 | 615279 | 15q22.31 AD |
| *MAP3K1 [MAPKKK1, MEKK, MEKK1]* | mitogen-activated protein kinase kinase kinase 1 | 46XY sex reversal 6 | 613762 | 5q11.2 AD |
| *MAPK1 [ERK, ERK2, MAPK2, PRKM1, PRKM2, p41mapk]* | mitogen-activated protein kinase 1 | Noonan syndrome 13 | 619087 | 22q11.22 AD |
| *MAPK3 [ERK1, PRKM3, p44erk1, p44mapk]* | mitogen-activated protein kinase 3 | n.a. | n.a. | 16p11.2 AD* |
| *MRAS [M-RAs, R-RAS3, RRAS3]* | muscle RAS oncogene homolog | Noonan syndrome 11 | 618499 | 3q22.3 AD |
| *NF1 [WSS, NFNS, VRNF]* | neurofibromin 1 | Neurofibromatosis, familial spinal; Neurofibromatosis, type 1; Neurofibromatosis-Noonan syndrome; | 162210; 162200; 601321; 193520 | 17q11.2 AD |

| | | | | |
|---|---|---|---|---|
| | | Watson syndrome | | |
| *NRAS [N-ras]* | NRAS proto-oncogene, GTPase | Noonan syndrome 6 | 613224 | 1p13.2 AD |
| *PPP1CB [MP, PP-1B, PP1B, PP1beta, PP1c, PPP1beta]* | protein phosphatase 1 catalytic subunit beta | Noonan syndrome-like disorder with loose anagen hair 2 | 617506 | 2p23.2 AD |
| *PTPN11 [BPTP3, NS1, PTP2C, SH-PTP2, SHP-2, SHP2]* | protein tyrosine phosphatase non-receptor type 11 | LEOPARD syndrome 1; Metachondromatosis; Noonan syndrome 1 | 151100; 156250; 163950 | 12q24.13 AD |
| *RAF1 [CRAF, Raf-1, c-Raf]* | Raf-1 proto-oncogene, serine/threonine kinase | Cardiomyopathy, dilated, 1NN; LEOPARD syndrome 2; Noonan syndrome 5 | 615916; 611554; 611553 | 3p25.2 AD |
| *RIT1 [MGC125864, MGC125865, RIBB, RIT, ROC1]* | Ras like without CAAX 1 | Noonan syndrome 8 | 615355 | 1q22 AD |
| *RRAS2 [TC21]* | RAS related 2 | Noonan syndrome 12 | 618624 | 11p15.2 AD |
| *SHOC2 [KIAA0862, SOC-2, SOC2, SUR-8, SUR8]* | SHOC2 leucine rich repeat scaffold protein | Noonan syndrome-like with loose anagen hair 1 | 607721 | 10q25.2 AD |
| *SOS1 [GF1, GINGF, HGF]* | SOS Ras/Rac guanine nucleotide exchange factor 1 | Fibromatosis, gingival, 1; Noonan syndrome 4 | 135300; 610733 | 2p22.1 AD |
| *SOS2* | SOS Ras/Rho guanine nucleotide exchange factor 2 | Noonan syndrome 9 | 616559 | 14q21.3 AD |
| *SPRED1 [FLJ33903, PPP1R147]* | sprouty related EVH1 domain containing 1 | Legius syndrome | 611431 | 15q14 AD |
| *SPRED2 [Spred-2, FLJ21897, FLJ31917,]* | sprouty related EVH1 domain containing 2 | Noonan syndrome 14 | 619745 | 2p14 AR |

**MAPK3* gene's inheritance mode is yet to be determined. In this study, autosomal dominant inheritance mode was considered. AD, autosomal dominant; AR, autosomal recessive; n.a., not available

**Supplementary Table 3. Variant pre-filtering criteria after annotation with Ensembl VEP.**

| VEP annotations | Inclusion and exclusion criteria |
|---|---|
| MAX_AF | Exclude >0.01 |
| BIOTYPE | Include “protein_coding” |
| Canonical | Include “yes” |
| INTRON | Include “-“ |
| EXON | Exclude “-“ |
| Consequence | Include when at least one of the following is present: "frameshift_variant", "missense_variant", "stop_gained", "stop_lost", "start_lost", "splice_region_variant", "splice_acceptor", "splice_donor"<br><br>Exclude "inframe_deletion", "inframe_duplication", "inframe_insertion" (all variants including any such consequence) |
| IMPACT | Exclude “LOW” |
| SIFT | Exclude “tolerated” * |
| POLYPHEN | Exclude “benign” * |
| CADD_PHRED (all respecitive columns) | Exclude ≤10 |
| CLINSIG | Exclude benign and likely benign (if combined with other classifications then retain the variant) |
| ClinVar_CLNSIG | Exclude benign and likely benign (if combined with other classifications then retain the variant) |

*Exclude only variants where both SIFT and POLYPHEN criteria were met.

**Supplementary Table 4. PCR and Sanger sequencing primers, product lengths, and PCR annealing conditions.**

| Gene | Variant | Forward primer (5´-3´)<br>Reverse primer (5´-3´)<br>Sequencing primer (5´-3´) | Size (bp) | Annealing temperature (°C) | Note |
|---|---|---|---|---|---|
| *CHEK1* | c.1036C>T<br>p.Q346Ter | TGAGAACTTGTGTGTGATATAA<br>TATTTTGCATGAAGGCCTCCCT<br>GTGAAGTACTCCAGTTCTCAGC | 420 | Touchdown<br>(58.4 to 47.4) | |
| *KIF7* | c.434A>C<br>p.Y145S | GGGTATAGAAGCATGTACTGAA<br>CTGCCTTCTCCATCCTAGAG<br>AGTTCCTCACCAACATTCCCGC | 495 | 53.4 | |
| *LZTR1* | c.848G>A<br>p.R283Q | CCTGGGCTAGTCACCGTAAG<br>TCCTGGTAGCTGTCTGGAAC<br>CCTGGGCTAGTCACCGTAAG | 438 | 56.5 | |
| *LZTR1* | c.891T>A<br>p.Y297Ter | CCCCTGTCCCAGCATTGATT<br>CCTTGCTCCTGGTAGCTGTC<br>CCCCTGTCCCAGCATTGATT | 363 | 69 | Q5 HiFi (NEB) |
| *LZTR1* | c.509G>A<br>p.R170Q | GCCAGATTCTGCTCCACCTT<br>TCAGGGTCCTCATCTGGGAG<br>GCCAGATTCTGCTCCACCTT | 288 | 69 | Q5 HiFi (NEB) |
| *LZTR1* | c.540del<br>p.T181Rfs Ter19 | GCCTCTGTGGCTGTCTGAAT<br>CATCCCCCTCCCAAGAAACC<br>GCCTCTGTGGCTGTCTGAAT | 450 | 68.0 | Q5 HiFi (NEB) |
| *MAP2K1* | c.635G>C<br>p.S212T | TCTCTTCCCCAATCTACCTGTG<br>CTGGGTTTGTGGATCTGAAGAC<br>TCTCTTCCCCAATCTACCTGTG | 511 | 57.1 | |
| *MRAS* | c.359C>T<br>p.P120L | GGCTGTGCTATGCCTGAGAT<br>CTCCTGACCCCCTCTACACA<br>GGCTGTGCTATGCCTGAGAT | 228 | 68.0 | Q5 HiFi (NEB) |
| *NF1* | c.4348G>T<br>p.A1450S | TAGCAGATGTCTTGTGTTATGA<br>CTGAGTAAGTGGCAAGAAAATT<br>TAGCAGATGTCTTGTGTTATGA | 447 | 51.4 | |
| *PTPN11* | c.922A>G<br>p.N308D | CATCAGGCAGTGTTCACGTTAC<br>TTTGGGCTTTGAATTGTTGCAC<br>TTTGGGCTTTGAATTGTTGCAC | 418 | 53.5 | |
| *PTPN11* | c.1510A>G<br>p.M504V | GTTGGCAAGTGAGGGAATCC<br>CCAAGAGGCCTAGCAAGAGA<br>GTTGGCAAGTGAGGGAATCC | 432 | Touchdown<br>(60.0 to 50.0) | |
| *PTPN11* | c.362A>C<br>p.E121A | TGTGTTTAGGAGAGCTGACTGT<br>CCAAAGATTTGGGTCACCAGAC<br>TGTGTTTAGGAGAGCTGACTGT | 493 | 66.0 | Q5 HiFi (NEB) |
| *PTPN11* | c.172A>G<br>p.N58D | AAAATCCGACGTGGAAGATGAGA<br>GTCATACACAGACCGTCATGC<br>AAAATCCGACGTGGAAGATGAGA | 364 | 67.0 | Q5 HiFi (NEB) |
| *PTPN11* | c.518G>T<br>p.R173L | TGTGTTTAGGAGAGCTGACTGT<br>CCAAAGATTTGGGTCACCAGAC<br>TGTGTTTAGGAGAGCTGACTGT | 493 | 66.0 | Q5 HiFi (NEB) |

| | | | | | |
|---|---|---|---|---|---|
| *SOS1* | c.1310T>C<br>p.I437T | TCAATGCTGCCATCCAATTGT<br>GCATGTCGGTTTTATAGTCAGC<br>TCAATGCTGCCATCCAATTGT | 415 | 53.8 | |
| *SOS1* | c.642A>C<br>p.Q214H | TGGAGTACATGGAGAATTCTGTG<br>AACTGACGAAGAGCCTTCCA<br>TGGAGTACATGGAGAATTCTGTG | 403 | 58.6 | |
| *SOS2* | c.26A>G<br>p.E9G | ATAACAAAACCTTCCTTCCGGG<br>TATTCGGGATAATGAGCGCC<br>TGAGAAACGTCCTTCAGAATGC | 982 | 49.8 | DMSO (6%) |
| *SPRED1* | c.973C>T<br>p.R325Ter | TCTGTACTCTTGTGGGGATGAG<br>GGCACATTCTCAAAGGGACG<br>TCTGTACTCTTGTGGGGATGAG | 404 | 56.3 | |
| *TP63* | c.1283C>T<br>p.P428L | TCCTTCTGCTCACTTCCATAGG<br>ATGGACCAGGCATATAGAGACG<br>TCCTTCTGCTCACTTCCATAGG | 447 | 57.1 | |

DMSO, dimethyl sulfoxide; n.a., not available; NEB, New England Biolabs; Q5 HiFi, Q5® High-Fidelity DNA Polymerase

**Supplementary Table 5. Assessment of variant pathogenicity in the ESTAND cohort.**

| No | cDNA & protein change | Variant type | gnomAD v2.1.1 (all, NFE, EST) | CADD score | ACMG* | ClinVar (Dec 1st, 2023) | ClinVar entries and/or Pubmed | Final decision |
|---|---|---|---|---|---|---|---|---|
| 1 | *PTPN11* c.922A>G p.N308D | missense | $1.19\times10^{-5}$ 0.00 0.00 | 24.5 | PS3, PS4, PM1, PM6, PM5, PP1, PP2, PP3 | P (FDA) | ClinVar: reported in ~60 cases with RASopathies. | P |
| 2 | *PTPN11* c.1510A>G p.M504V | missense | $3.98\times10^{-6}$ $8.79\times10^{-6}$ 0.00 | 27.1 | PS3, PM1, PM6, PP2, PP3 | P (FDA) | ClinVar: reported in ~30 cases with RASopathies. | P |
| 3 | *SOS1* c.642A>C p.Q214H | missense | n.a. | 21.0 | PM2, PP2 | n.a. | ClinVar: SOS1 c.642A>T (p.Q214H) reported as VUS without phenotype | LP |
| 4 | *SOS1* c.406T>C p.Y136H | missense | n.a. | 28.0 | PM2, PM6, PP2, PP3 | LP | Previously published ESTAND case[5]; also reported in ClinVar. | LP |
| 5 | *SOS1* c.1310T>C p.I437T | missense | n.a. | 25.9 | PM1, PM2, PM5, PP2, PP3 | LP/P | ClinVar: 12 cases, including 6 diagnosed with NS. | P |
| 6 | *SOS2* c.26A>G p.E9G; | missense | n.a. | 24.8 | PM2 | n.a. | n.a. | LP |
| | *KIF7#* c.434A>C p.Y145S; | missense | $1.28\times10^{-5}$ $3.29\times10^{-5}$ 0.00 | 27.5 | PM2, PP3** | Conflicting (LP/P, VUS) | ClinVar: cerebellar ataxia and other conditions (LP), acrocallosal syndrome (P or VUS) | LP |
| | *CHEK1* c.1036C>T p.Q346Ter | stop gained | $3.89\times10^{-5}$ $7.74\times10^{-5}$ $8.28\times10^{-4}$ | 38.0 | PM2** | LP | ClinVar: reported in one case without phenotype data | LP |

| | | | | | | | | |
|---|---|---|---|---|---|---|---|---|
| 7 | *LZTR1* c.848G>A p.R283Q | missense | 0.00 0.00 0.00 | 32.0 | PS3, PM6, PP2 | Conflicting (10 LP/P, two VUS) | ClinVar: 12 cases, including 6 diagnosed with NS. | P |
| 8 | *NF1* c.4348G>T p.A1450S | missense | n.a. | 27.9 | PM1, PM2, PP2, PP3 | VUS | ClinVar: one case with cardiovascular phenotype and hereditary cancer syndrome | LP |
| 9 | *SPRED1* c.973C>T p.R325Ter; | stop gained | n.a. | 38.0 | PS3, PM2, PP3 | P | ClinVar: three cases with Legius syndrome or cardiovascular phenotype. | P |
| | *TP63* c.1283C>T p.P428L | missense | n.a. | 26.8 | PM2, PP2, PP3, PP4 | n.a. | n.a. | LP |
| 10 | *MAP2K1* c.635G>C p.S212T | missense | n.a. | 27.3 | PM2, PP2, PP3 | n.a. | n.a. | LP |

* Based on RASopathy-related ACMG guidelines[16].
** Based on the general ACMG guidelines[29].
#*KIF7* variants have been reported as causative heterozygous mutations following the autosomal dominant inheritance pattern among patients diagnosed with hydrolethalus and acrocallosal syndromes[30].
ACMG, American College of Medical Genetics and Genomics; AD, autosomal dominant; AR, autosomal recessive; EST, Estonians; FDA, Food and Drug Administration; LP, likely pathogenic; n.a., not available; NFE, Non-Finnish Europeans; NS, Noonan syndrome; P, pathogenic; VUS, variant of uncertain significance

**Supplementary Table 6. Clinical parameters of the GEMINI cohort subgroups.**

| | Clinical subgroups (median values; n for subjects with data) | | | |
|---|---|---|---|---|
| Parameter (unit) | All infertile men | NOA | Oligozoospermia | Fertile controls |
| Subjects (n) | 1,416 | 1,119 | 297 | 317 |
| Age (years) | 35 (191) | 35 (191) | n.a. | n.a. |
| Sperm concentration ($\times 10^6$/mL) | 0.003 (532) | 0.0 (249) | 0.2 (283) | 137.4 (124) |
| Semen volume (mL) | 2.9 (247) | 2.6 (140) | 3.0 (107) | 3.5 (124) |
| Total sperm counts ($\times 10^6$ per ejaculate) | 0.009 | 0.0 | 0.6 | 480.9 |
| Sperm motility (A+B, %) | 9.1 (120) | 0.0 (29) | 15.9 (88) | 56.0 (124) |
| FSH (IU/L) | 16.7 (576) | 17.9 (519) | 8.2 (47) | 3.0 (90) |
| LH (IU/L) | 7.2 (324) | 7.2 (276) | 7.2 (44) | 3.6 (90) |
| Testosterone (nmol/L) | 13.0 (315) | 13.0 (265) | 13.0 (47) | 17.2 (90) |
| Left testis volume (mL) | 11.1 (362) | 10.0 (318) | 15.0 (42) | n.a. |
| Right testis volume (mL) | 12.0 (366) | 10.0 (318) | 17.0 (45) | n.a. |
| Total TV (mL) | 22.0 (368) | 20.0 (320) | 32.0 (45) | n.a. |
| Total TV < 30 mL (n, %) | 251, 68.2% | 234, 73.1% | 15, 33.3% | n.a. |
| Cryptorchidism (n, %) | 31, 2.2% | 24, 2.1% | 7, 2.4% | n.a. |

Data are shown as median, unless indicated otherwise. The number of men with available data for the given parameter is shown in the brackets.
FSH, follicle-stimulating hormone; LH, luteinizing hormone; NOA, non-obstructive azoospermia

**Supplementary Table 7. Assessment of variant pathogenicity in the GEMINI cohort.**

| No | Gene; cDNA, protein change | gnomAD v2.1.1 (all, NFE) | CADD score | ACMG[a] | ClinVar (n, cases) Dec 1st, 2023 | Final decision |
|---|---|---|---|---|---|---|
| 11 | *LZTR1* c.891T>A p.Y297Ter | 0.00 0.00 | 33.0 | PVS1, PM2, PP3, PP5 | P (1) | P |
| 12 | *LZTR1* c.509G>A p.R170Q | $1.99\times10^{-5}$ $2.64\times10^{-5}$ | 34.0 | PS3, PM1, PM3, PP2, PP3 | LP (2) / P (2) | LP |
| 13 | *LZTR1* c.540del p.T181RfsTer19 | n.a. | n.a. | PVS1, PS1, PP5 | P (1) | P |
| 14 | *PTPN11* c.362A>C p.E121A | n.a. | 24.9 | PM2, PP2, PP3 | n.a. | LP |
| 15 | *PTPN11* c.172A>G p.N58D | n.a. | 26.6 | PS2, PM1, PM2, PM5, PP2, PP3 | P (19) | P |
| 16 | *PTPN11* c.518G>T p.R173L | $1.59\times10^{-5}$ 0.00 | 24.3 | PM2, PM5, PP2, PP3 | n.a. | LP |
| 17 | *MRAS* c.359C>T p.P120L | n.a. | 28.0 | PM2, PP3 | n.a. | LP |

[a] Based on RASopathy-related ACMG guidelines[16].
ACMG, American College of Medical Genetics and Genomics; NFE, Non-Finnish Europeans; LP, likely pathogenic; P, pathogenic; n.a., not available.

**Supplementary Table 8**. **Testicular histopathology analysis of Case 1.**

| | **Case 1: PTPN11 p.Asn308Asp (Pathogenic, FDA expert panel)** |
|---|---|
| Uniform morphology | No |
| Tubules contain germinative cells | Yes |
| Tubules contain only Sertoli cells | Yes |
| Tubules contain cells from all stages of spermatogenesis | 40% |
| Tubules with spermatocytic arrest | 10% |
| Tubules contain only spermatogonia and Sertoli cells | 2% |
| Tubules with only Sertoli cells | 20% |
| Partially hyalinized tubules | 18% |
| Fully hyalinized tubules | 10% |
| Tubular lamina propria | Moderately-severely thickened |
| The number of Leydig cells | Severely hyperplastic |
| Testicular germ cell neoplasia *in situ* | None |

**Supplementary Table 9.** Statistical modeling of digenic pathogenicity of the RASopathy-linked variant and incidental finding in another developmental gene in Cases 6 and 9.

| Case | Gene | Genomic change (hg38) | cDNA change, protein change | ACMG classif. | VarCoPP Score = probability of digenic effect | Confidence zone for digenic effect |
|---|---|---|---|---|---|---|
| 6 | *SOS2* | chr14:50231258-T/C | c.26A>G p.E9G | LP | 0.83 | 99% |
| | *KIF7* | chr15:89649836-T/G | c.434A>C p.T145S | LP | | |
| 9 | *SPRED1* | chr15:38351302-C/T | c.973C>T p.R325Ter | LP | 0.94 | 99.9% |
| | *TP63* | chr3:189872929-C/T | c.1283C>T p.P428L | LP | | |

Information and classification of individual variants is provided in **Supplementary Table 5.** Statistical modeling of digenic pathogenicity of disease-causing variant pairs was implemented at the ORVAL (the Oligogenic Resource for Variant AnaLysis) platform (https://orval.ibsquare.be). VarCoPP2.0 (Variant Combinations Pathogenicity Predictor 2.0) score indicates the probability (value between 0 and 1) that the combination of the variant pair is pathogenic[19-21]. Digenic Effect Predictor Cut-offs: candidate disease-causing ≥ 0.5; 99% confident prediction ≥ 0.647; 99.9% confident prediction ≥ 0.850.

ACMG, American College of Medical Genetics and Genomics; AD, autosomal dominant; AR, autosomal recessive; classif., classification; n.a., not available; LP, likely pathogenic; P, pathogenic

## REFERENCES TO ONLINE-ONLY MATERIALS

1. Punab M, Poolamets O, Paju P, et al. Causes of male infertility: a 9-year prospective monocentre study on 1737 patients with reduced total sperm counts. Hum Reprod. 2017;32(1):18-31. doi: 10.1093/humrep/dew284
2. World Health Organization. WHO laboratory manual for the examination and processing of human semen Sixth Edition. 2021.
3. Ehala-Aleksejev K, Punab M. The different surrogate measures of adiposity in relation to semen quality and serum reproductive hormone levels among Estonian fertile men. Andrology. 2015;3(2):225-34. doi: 10.1111/andr.12002
4. Kasak L, Punab M, Nagirnaja L, et al. Bi-allelic Recessive Loss-of-Function Variants in FANCM Cause Non-obstructive Azoospermia. Am J Hum Genet. 2018;103(2):200-212. doi: 10.1016/j.ajhg.2018.07.005.
5. Laan M, Kasak L, Timinskas K, et al. NR5A1 c.991-1G > C splice-site variant causes familial 46,XY partial gonadal dysgenesis with incomplete penetrance. Clin Endocrinol (Oxf). 2021;94(4):656-666. doi: 10.1111/cen.14381
6. Colpi GM, Francavilla S, Haidl G, et al. European Academy of Andrology guideline Management of oligo-astheno-teratozoospermia. Andrology. 2018;6(4):513-524. doi: 10.1111/andr.12502
7. Sulonen AM, Ellonen P, Almusa H, et al. Comparison of solution-based exome capture methods for next generation sequencing. Genome Biol. 2011;12(9):R94. doi: 10.1186/gb-2011-12-9-r94
8. Nagirnaja L, Lopes AM, Charng WL, et al. Diverse monogenic subforms of human spermatogenic failure. Nat Commun. 2022;13(1):7953. doi: 10.1038/s41467-022-35661-z
9. Li H, Durbin R. Fast and accurate short read alignment with Burrows-Wheeler transform. Bioinformatics. 2009;25(14):1754-1760. doi: 10.1093/bioinformatics/btp324
10. Faust GG, Hall IM. SAMBLASTER: fast duplicate marking and structural variant read extraction. Bioinformatics. 2014;30(17):2503-5. doi: 10.1093/bioinformatics/btu314
11. McKenna A, Hanna M, Banks E, et al. The Genome Analysis Toolkit: A MapReduce framework for analyzing next-generation DNA sequencing data. Genome Res. 2012;20(9):1297-1303. doi: 10.1101/gr.107524.110
12. Danecek P, Bonfield JK, Liddle J, et al. Twelve years of SAMtools and BCFtools. Gigascience. 2021;10(2):giab008. doi: 10.1093/gigascience/giab008
13. McLaren W, Gil L, Hunt SE, et al. The Ensembl Variant Effect Predictor. Genome Biol. 2016;17(1):122. doi: 10.1186/s13059-016-0974-4
14. Landrum MJ, Lee JM, Benson M, et al. ClinVar: improving access to variant interpretations and supporting evidence. Nucleic Acids Res. 2018;46(D1):D1062-D1067. doi: 10.1093/nar/gkx1153
15. Motta M, Fidan M, Bellacchio E, et al. Dominant Noonan syndrome-causing LZTR1 mutations specifically affect the Kelch domain substrate-recognition surface and enhance RAS-MAPK signaling. Hum Mol Genet. 2019;28(6):1007-1022. doi: 10.1093/hmg/ddy41240
16. Gelb BD, Cavé H, Dillon MW, et al. ClinGen's RASopathy Expert Panel Consensus Methods for Variant Interpretation. Genet Med. 2018;20(11):1334-1345. doi: 10.1038/gim.2018.3

17. Rodrigues EDS, Griffith S, Martin R,, et al. Variant-level matching for diagnosis and discovery: Challenges and opportunities. Hum Mutat. 2022;43(6):782-790. doi: 10.1002/humu.24359
18. Robinson JT, Thorvaldsdottir H, Turner D, Mesirov JP. igv.js: an embeddable JavaScript implementation of the Integrative Genomics Viewer (IGV). Bioinformatics. 2023;39(1):btac830. doi: 10.1093/bioinformatics/btac830
19. Renaux A, Papadimitriou S, Versbraegen N, et al. ORVAL: a novel platform for the prediction and exploration of disease-causing oligogenic variant combinations. Nucleic Acids Res. 2019;47(W1):W93-W98. doi: 10.1093/nar/gkz437
20. Versbraegen N, Gravel B, Nachtegael C, et al. Faster and more accurate pathogenic combination predictions with VarCoPP2.0. BMC Bioinformatics. 2023;24(1):179. doi: 10.1186/s12859-023-05291-3
21. Nachtegael C, Gravel B, Dillen A, et al. Scaling up oligogenic diseases research with OLIDA: the Oligogenic Diseases Database. Database (Oxford). 2022;2022:baac023. doi: 10.1093/database/baac023
22. Kõressaar T, Lepamets M, Kaplinski L, Raime K, Andreson R, Remm M. Primer3_masker: integrating masking of template sequence with primer design software. Bioinformatics. 2018;34(11):1937-1938. doi: 10.1093/bioinformatics/bty036
23. Ye J, Coulouris G, Zaretskaya I, Cutcutache I, Rozen S, Madden TL. Primer-BLAST: A tool to design target-specific primers for polymerase chain reaction. BMC Bioinformatics. 2012;13:134. doi: 10.1186/1471-2105-13-134
24. Andreson R, Reppo E, Kaplinski L, Remm M. GENOMEMASKER package for designing unique genomic PCR primers. BMC Bioinformatics. 2006;7:172. doi: 10.1186/1471-2105-7-172
25. Romano AA, Allanson JE, Dahlgren J, et al. Noonan Syndrome: Clinical Features, Diagnosis, and Management Guidelines. Pediatrics 2010;126(4):746-759. doi: 10.1542/peds.2009-3207
26. Bhambhani V, Muenke M. Noonan Syndrome. Am Fam Physician. 2014;89(1):37-43.
27. McLachlan RI, Rajpert-De Meyts E, Hoei-Hansen CE, de Kretser DM, Skakkebaek NE. Histological evaluation of the human testis--approaches to optimizing the clinical value of the assessment: mini review. Hum Reprod. 2007;22(1):2-16. doi: 10.1093/humrep/del279
28. Mahyari E, Guo J, Lima AC, et al. Comparative single-cell analysis of biopsies clarifies pathogenic mechanisms in Klinefelter syndrome. Am J Hum Genet. 2021;108(10):1924-1945. doi: 10.1016/j.ajhg.2021.09.001
29. Richards S, Aziz N, Bale S, et al. Standards and guidelines for the interpretation of sequence variants: a joint consensus recommendation of the American College of Medical Genetics and Genomics and the Association for Molecular Pathology. Genet Med. 2015;17(5):405-424. doi: 10.1038/gim.2015.30
30. Putoux A, Thomas S, Coene KL, et al. KIF7 mutations cause fetal hydrolethalus and acrocallosal syndromes. Nat Genet. 2011;43(6):601-606. doi: 10.1038/ng.826
